# Supplementary material for: Deletion of neuroligins from astrocytes does not detectably alter synapse numbers or astrocyte cytoarchitecture by maturity
Source: eLife. 2026 Aug 4;12:RP87589. doi: 10.7554/eLife.87589 (PMC13436961; doi:10.7554/eLife.87589)
Supplement: Supplementary file 2 [file elife-87589-fig4-data1.zip › Figure_4_-_source_data_7.pdf]

VGluT2 – Hippocampus

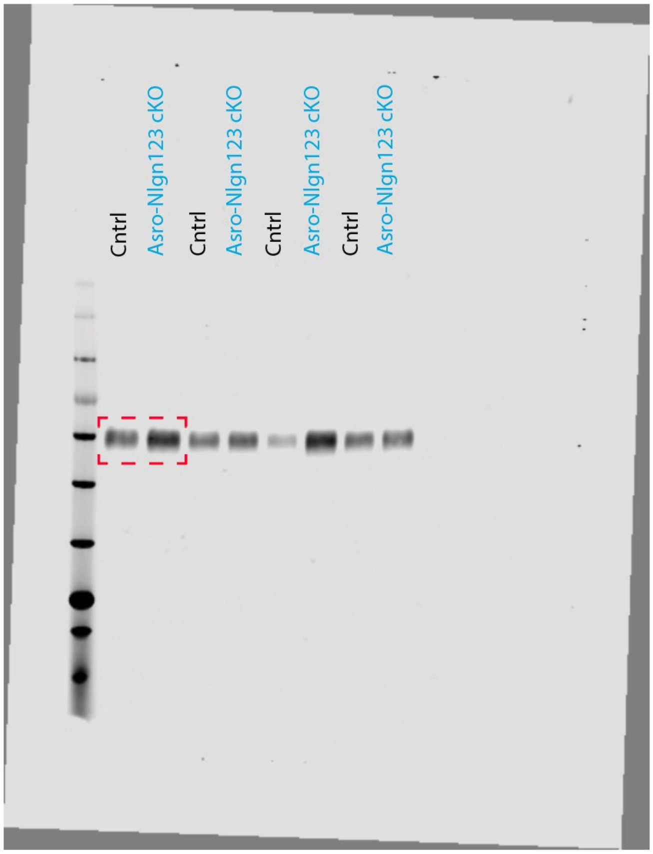

VGluT1 - Hippocampus

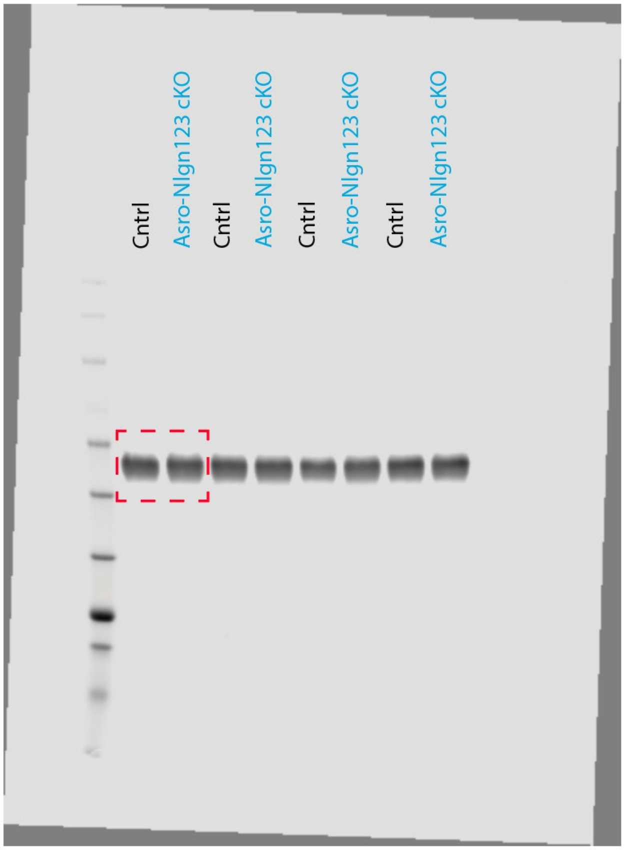

Syt1 – Hippocampus

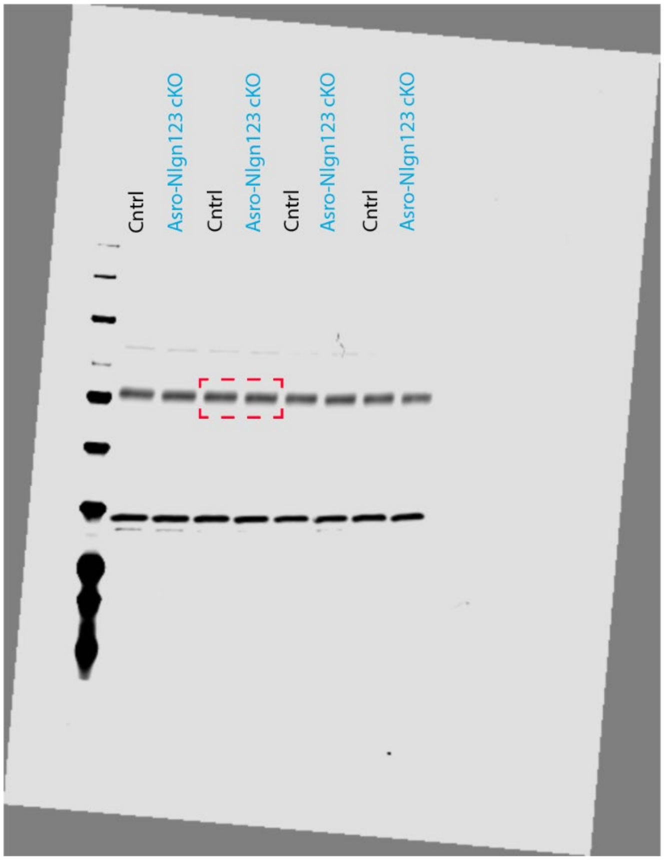

Syb2 – Hippocampus

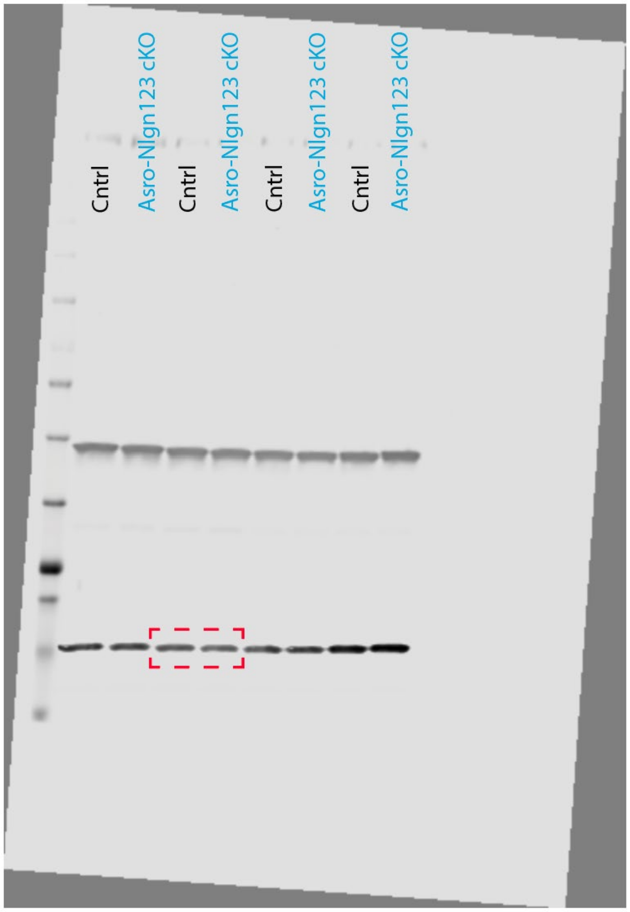

SNAP25 – Hippocampus

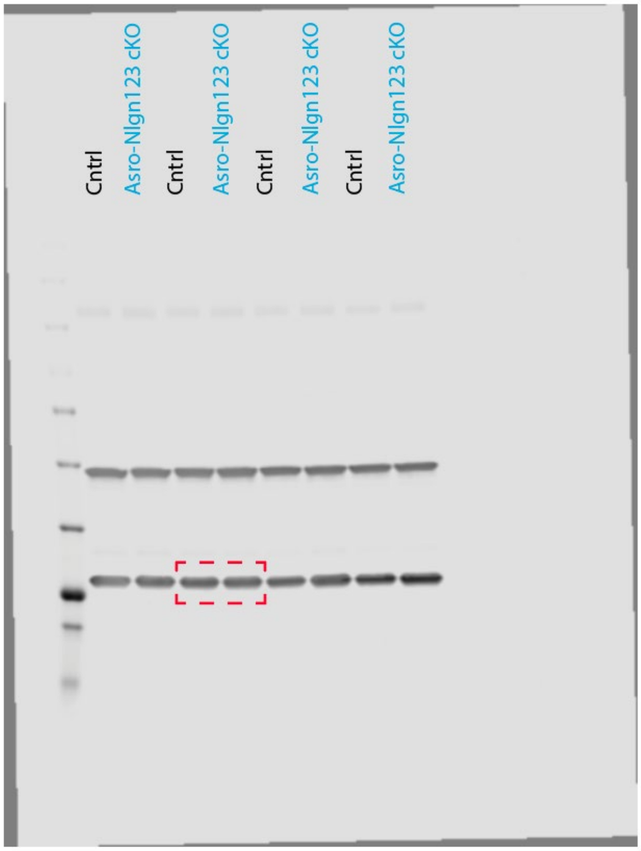

PSD95 – Hippocampus

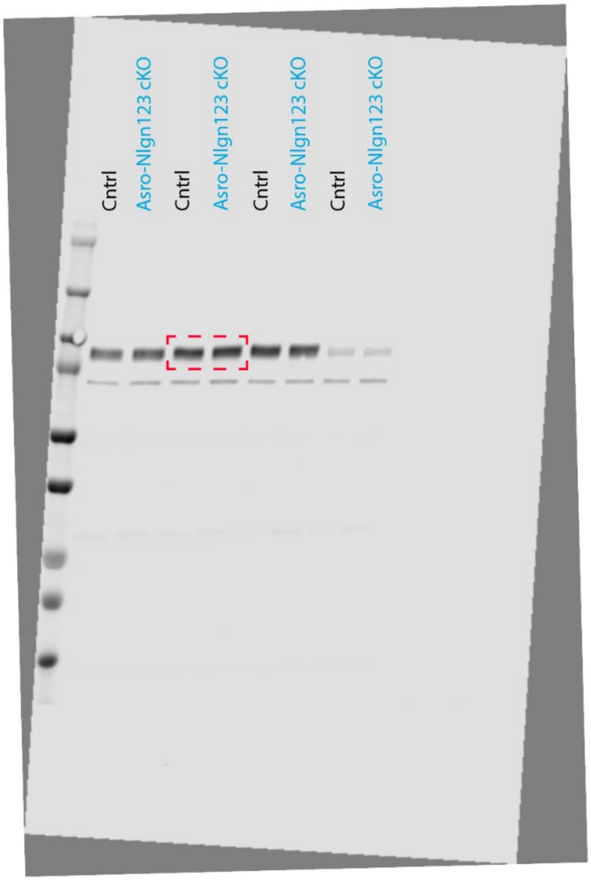

Mint1 – Hippocampus

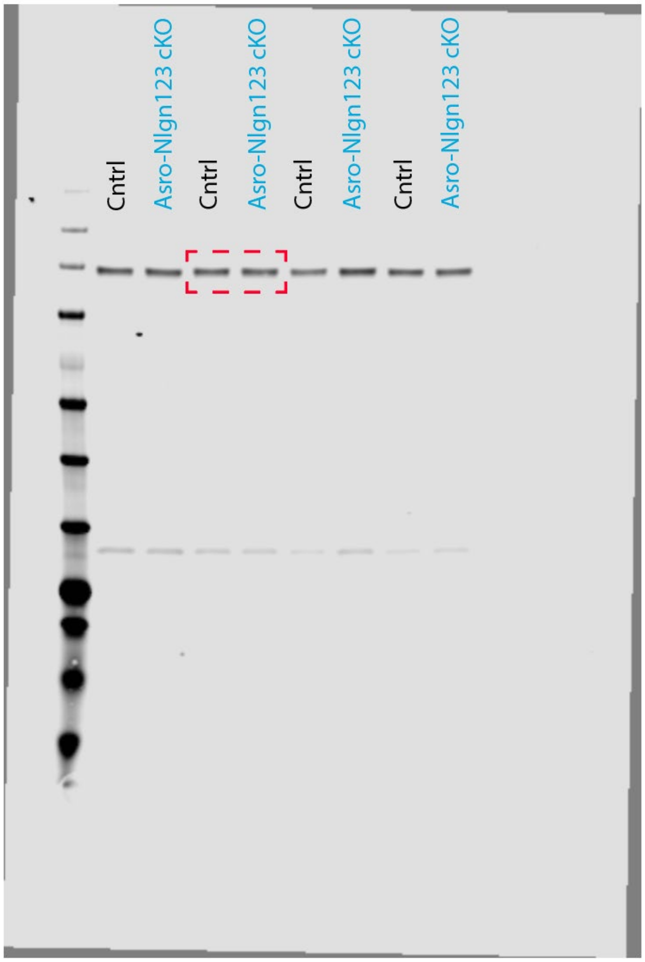

GRIP – Hippocampus

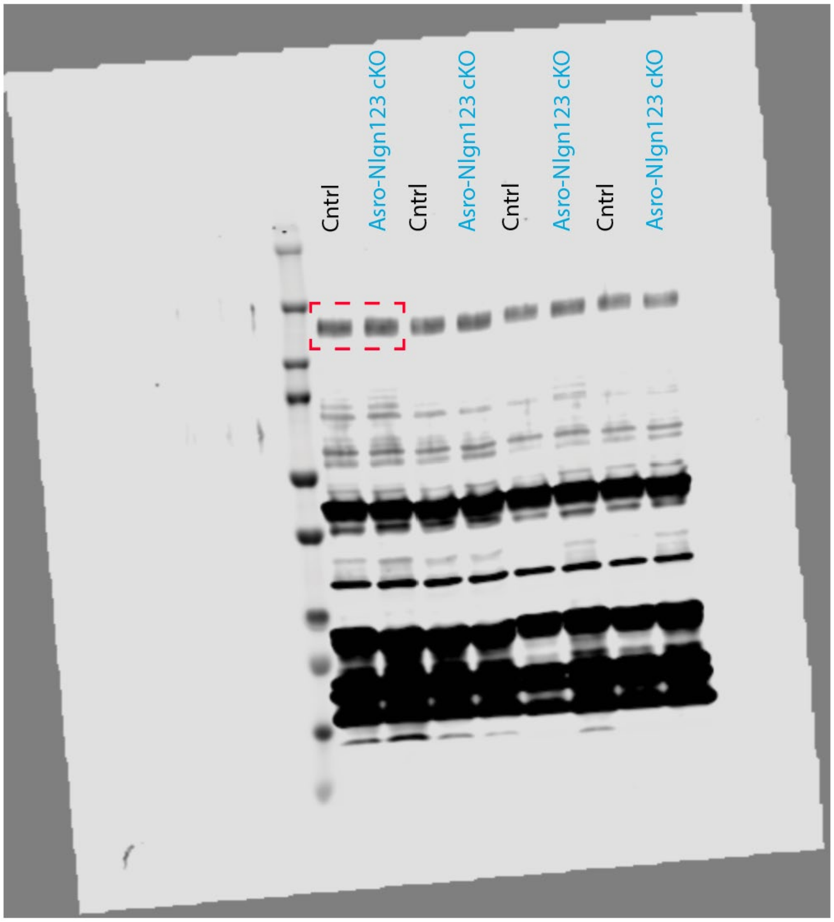

GluR2 – Hippocampus

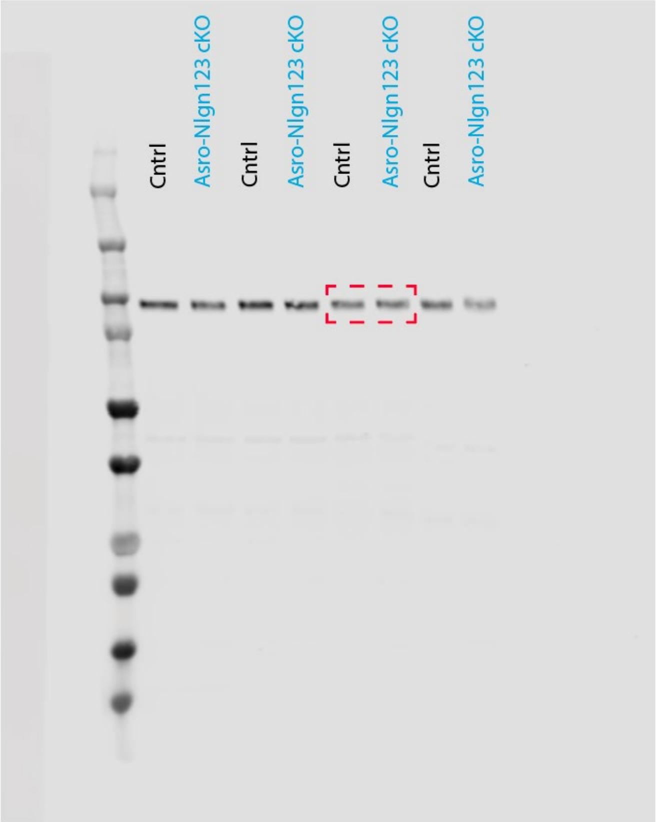

GluR1 – Hippocampus

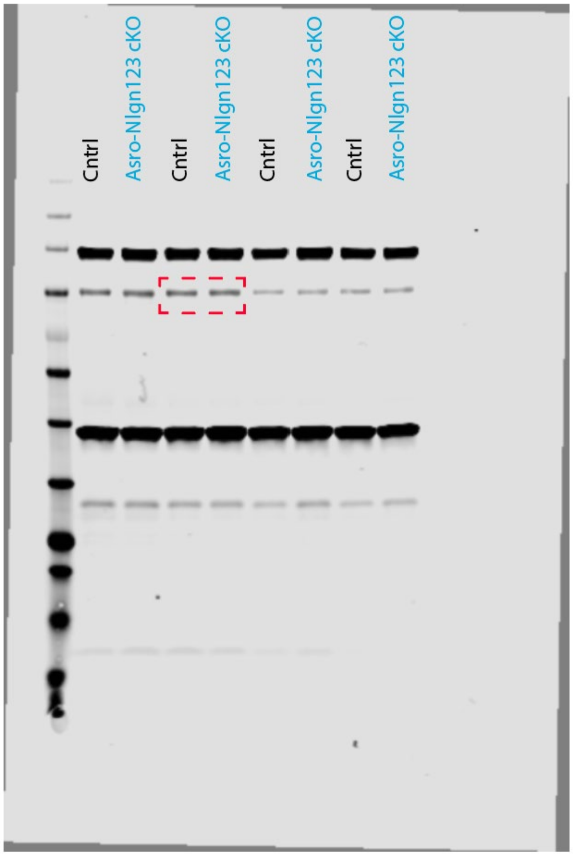

Gephyrin – Hippocampus

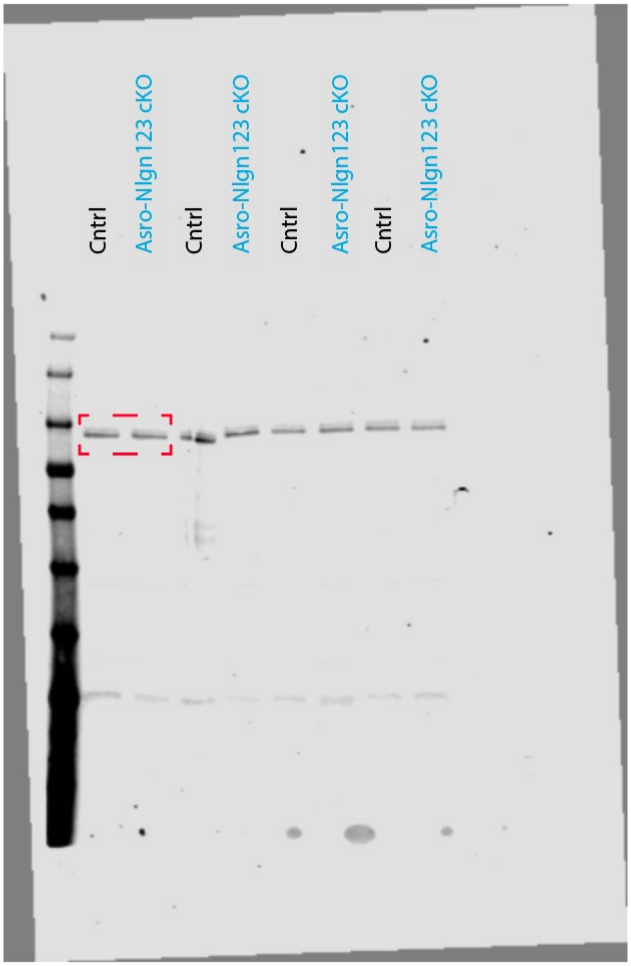

CASK – Hippocampus

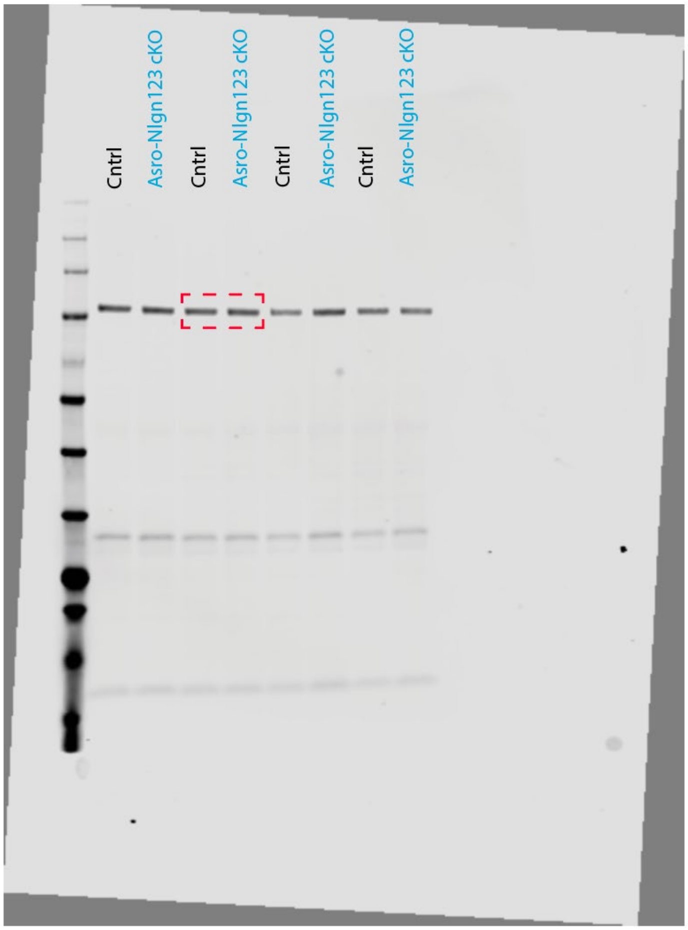

Calbindin – Hippocampus

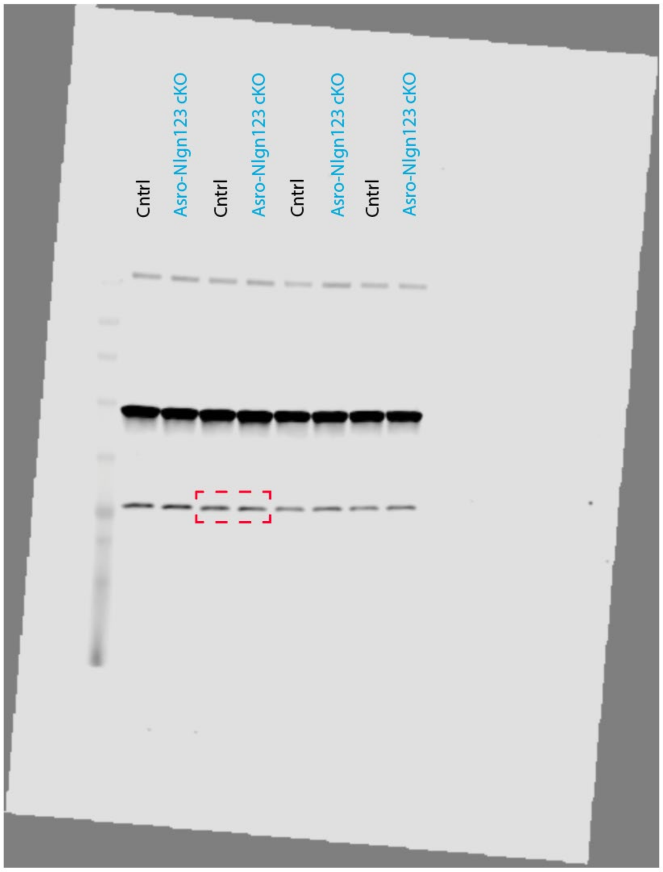

Actin – Hippocampus

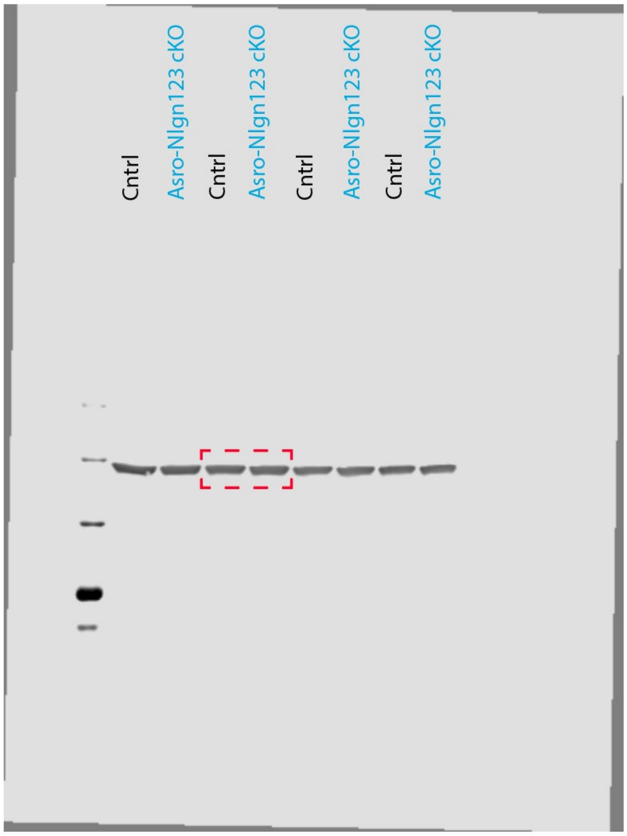

VGluT2 – Cortex

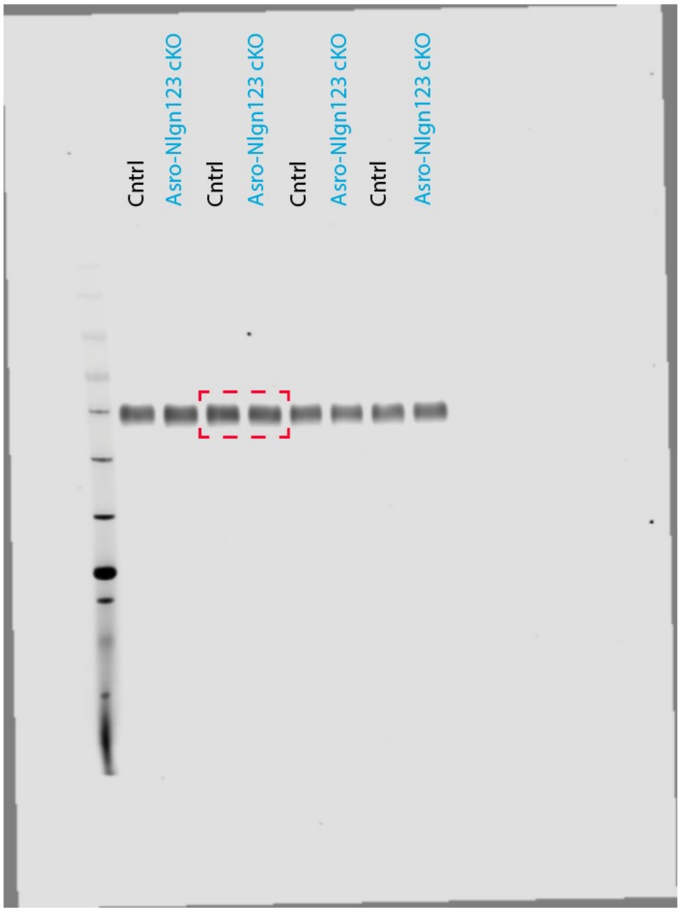

VGluT1 – Cortex

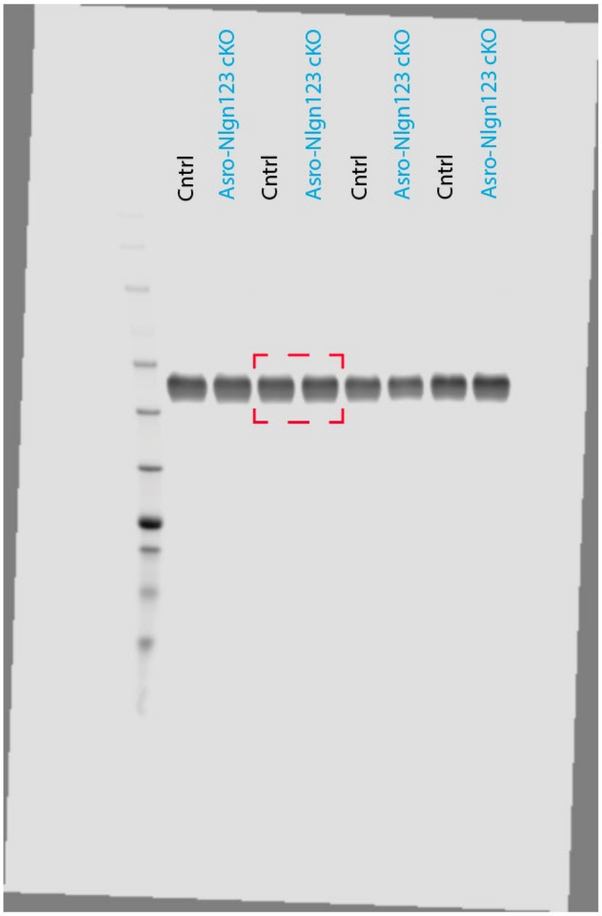

Syt1 – Cortex

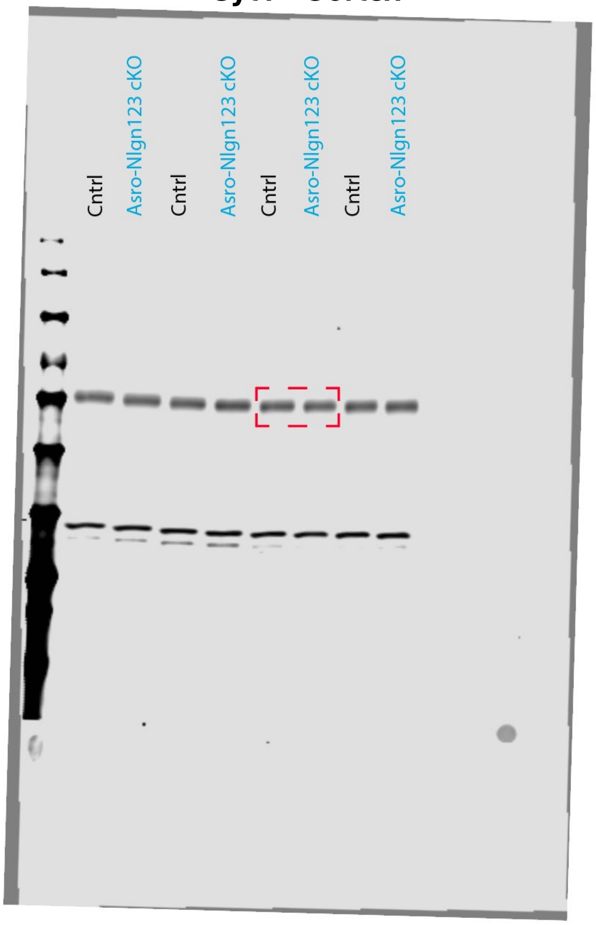

Syb2 – Cortex

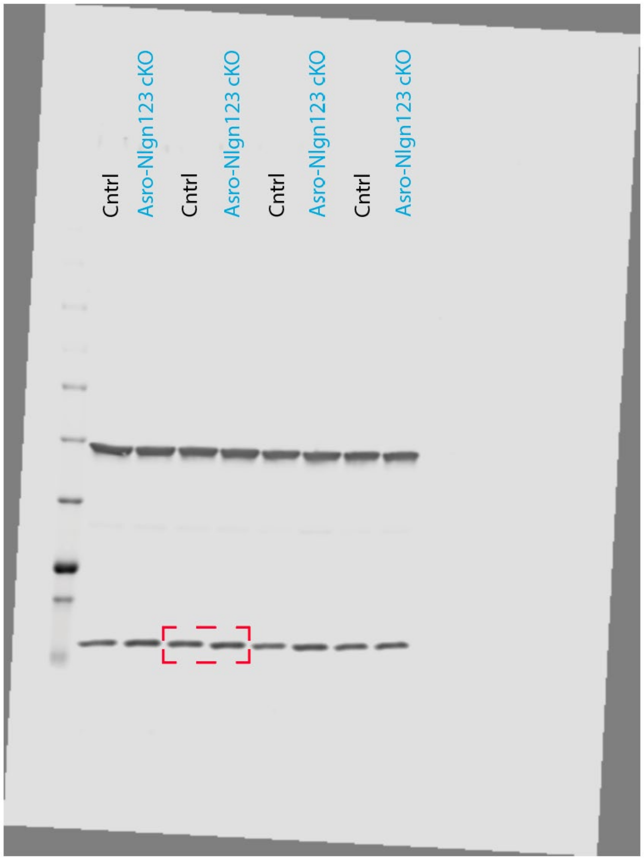

SNAP25 – Cortex

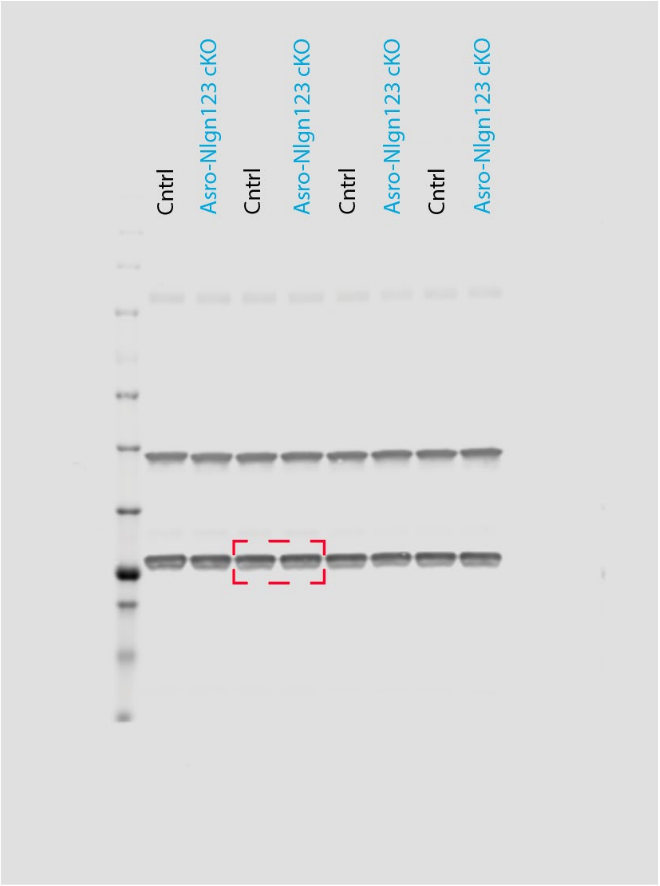

PSD95 – Cortex

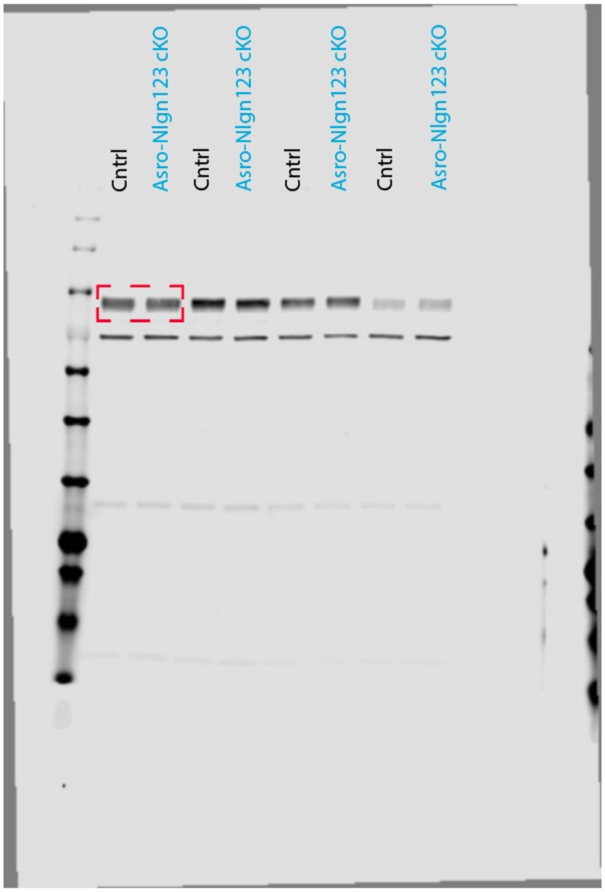

Mint1 – Cortex

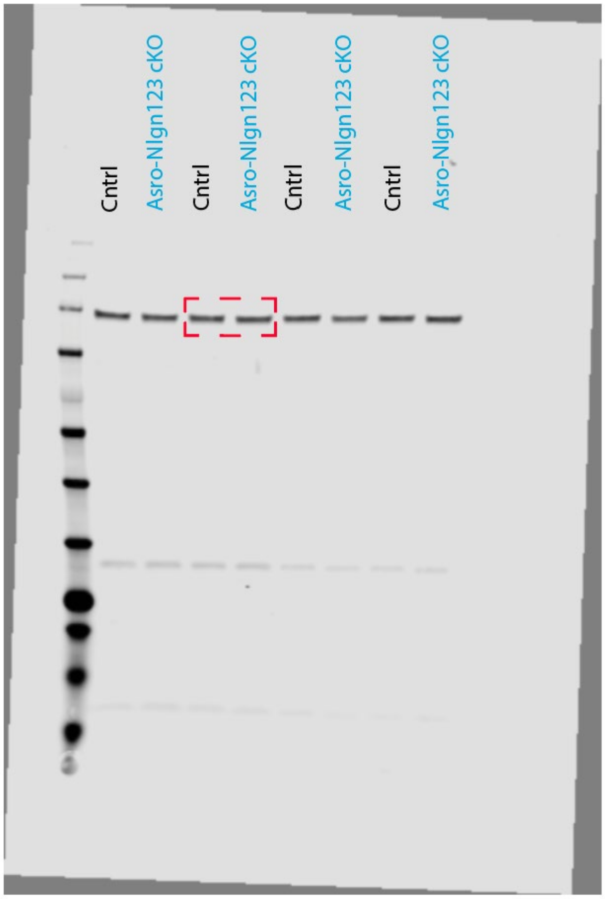

GRIP – Cortex

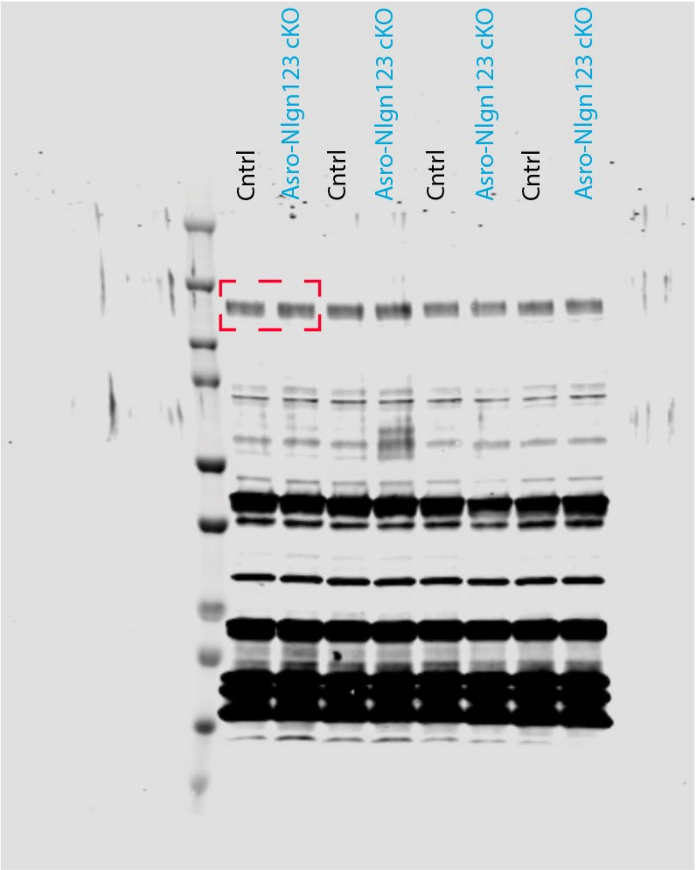

GluR2 – Cortex

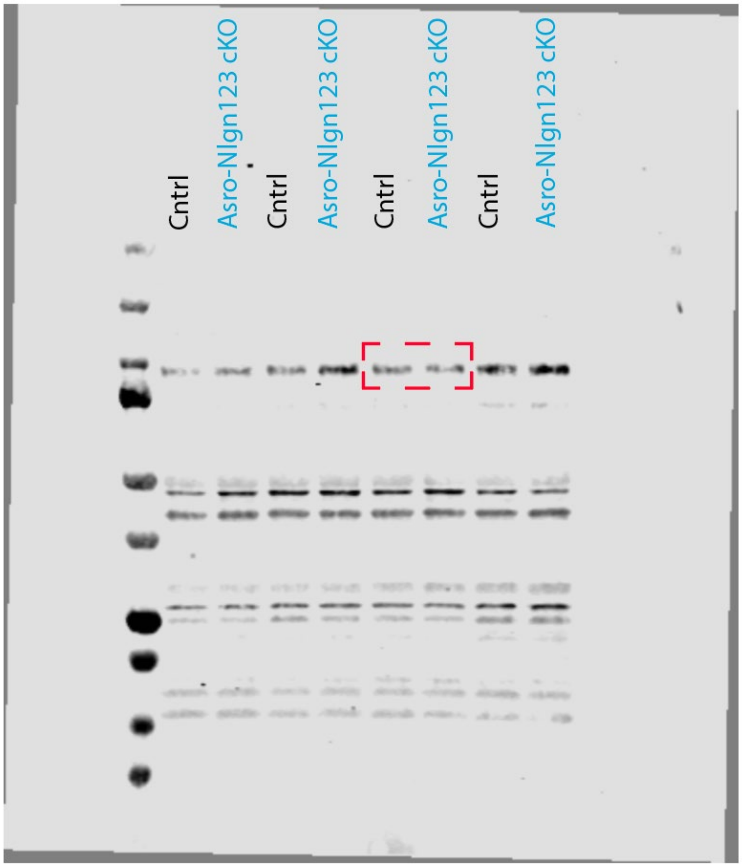

GluR1 – Cortex

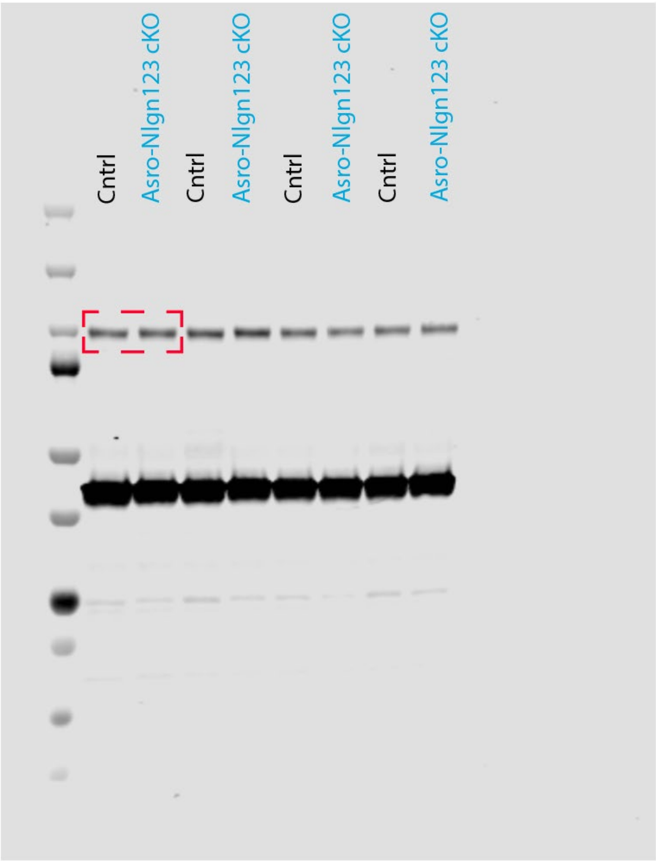

Gephyrin – Cortex

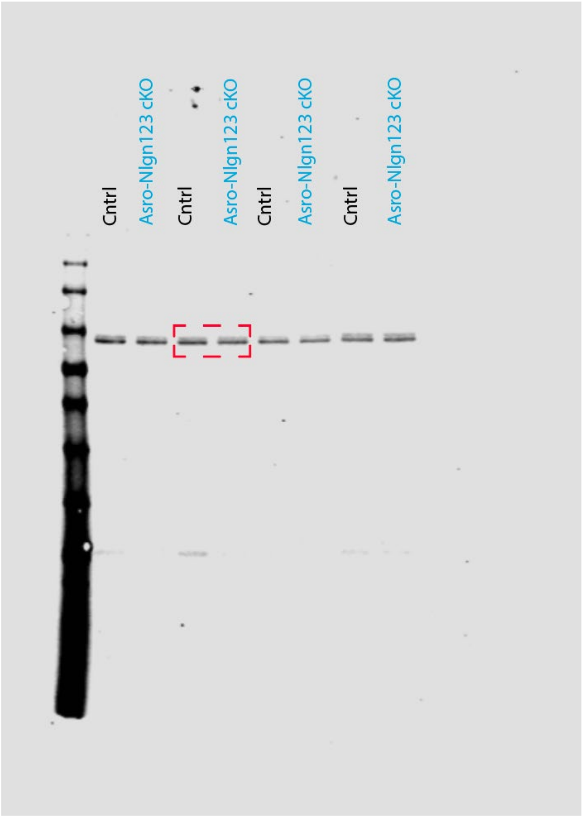

CASK – Cortex

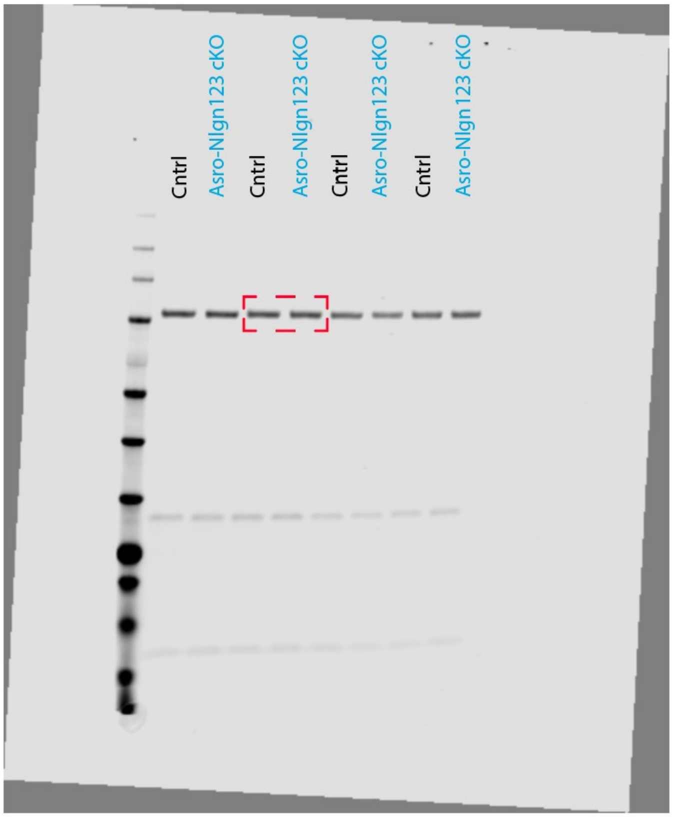

Calbindin – Cortex

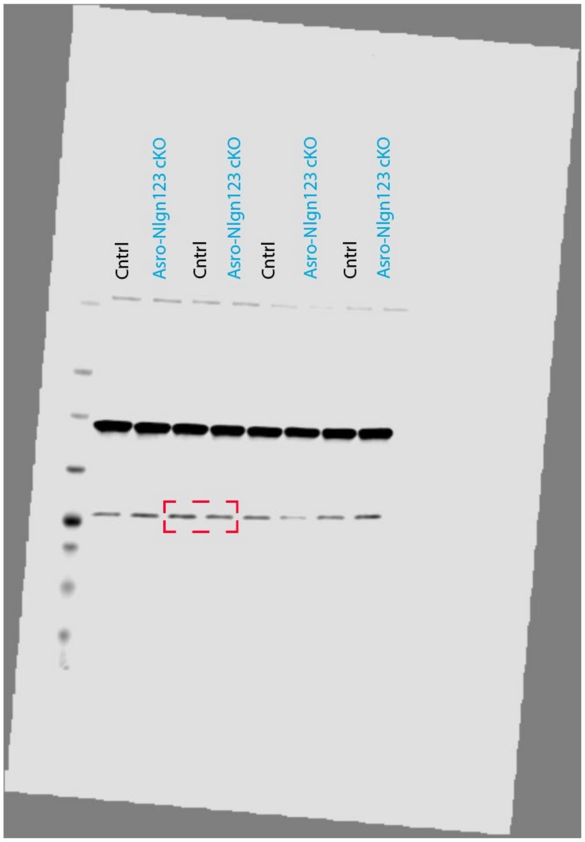

Actin – Cortex

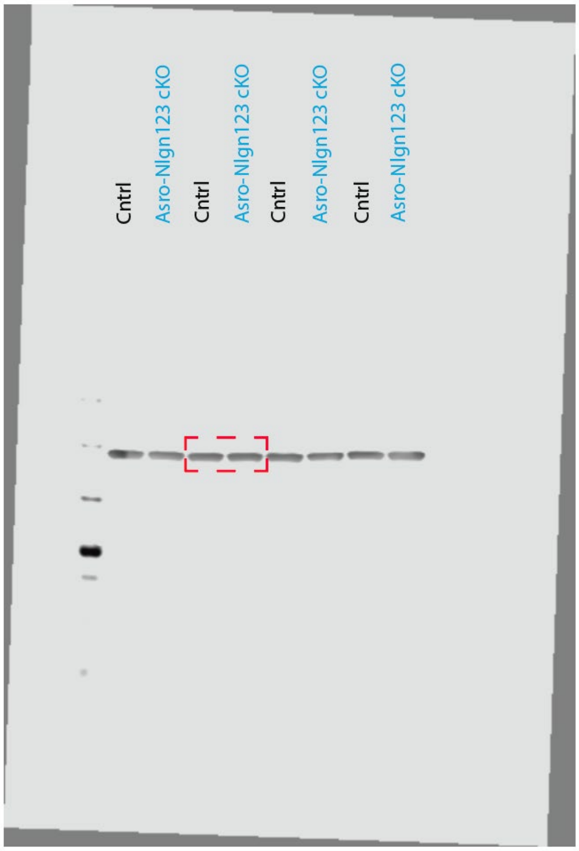

Figure 4, Source Data 7. Original membranes corresponding to Figure 4, panel G. Red dashed boxes indicate the regions cropped for the representative images shown in Figure 4.
