# Supplementary material for: Deletion of neuroligins from astrocytes does not detectably alter synapse numbers or astrocyte cytoarchitecture by maturity
Source: eLife. 2026 Aug 4;12:RP87589. doi: 10.7554/eLife.87589 (PMC13436961; doi:10.7554/eLife.87589)
Supplement: Supplementary file 2 [file elife-87589-fig4-data1.zip › Figure_4_-_source_data_5.pdf]

Nlgn1 – Hippocampus

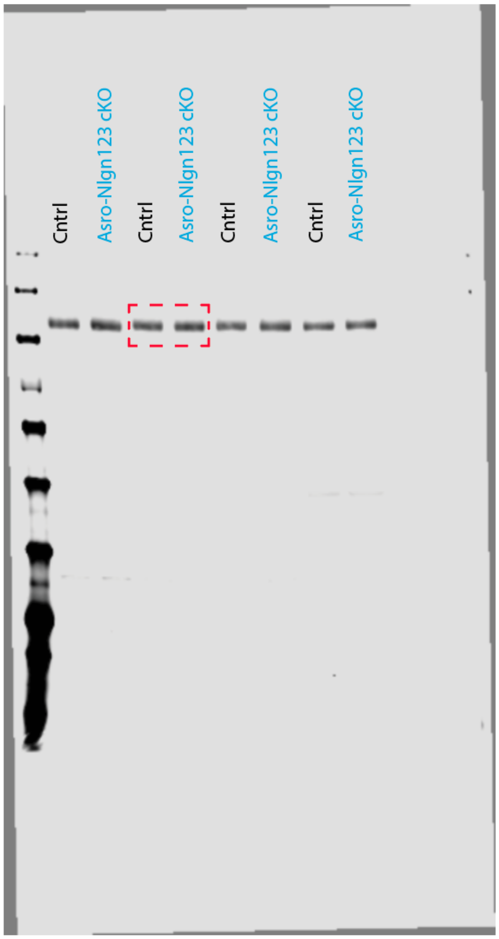

Nlgn2 – Hippocampus

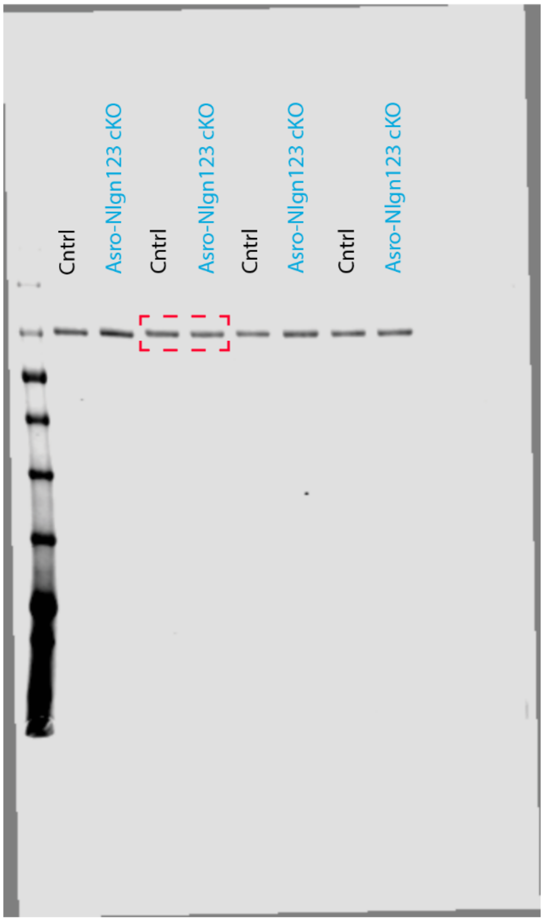

Nlgn3 – Hippocampus

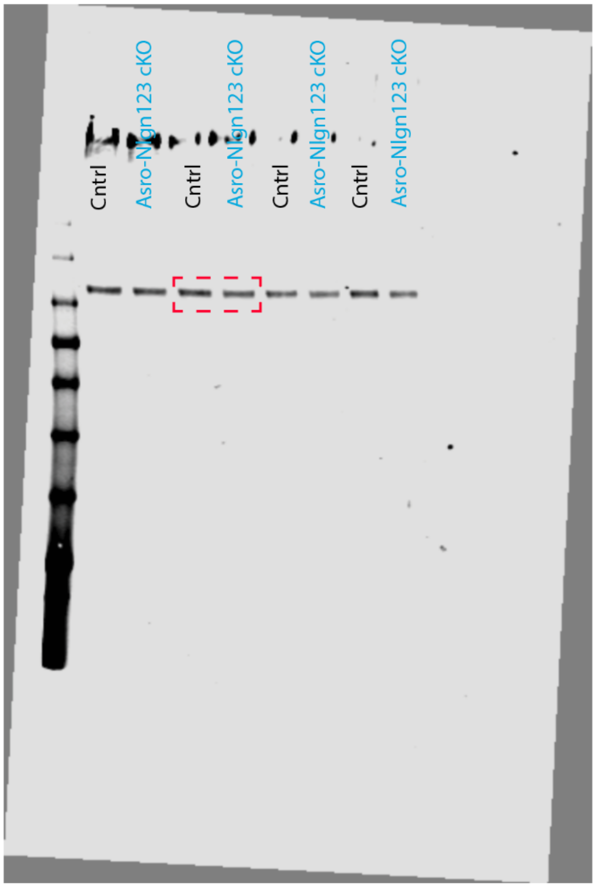

Actin – Hippocampus

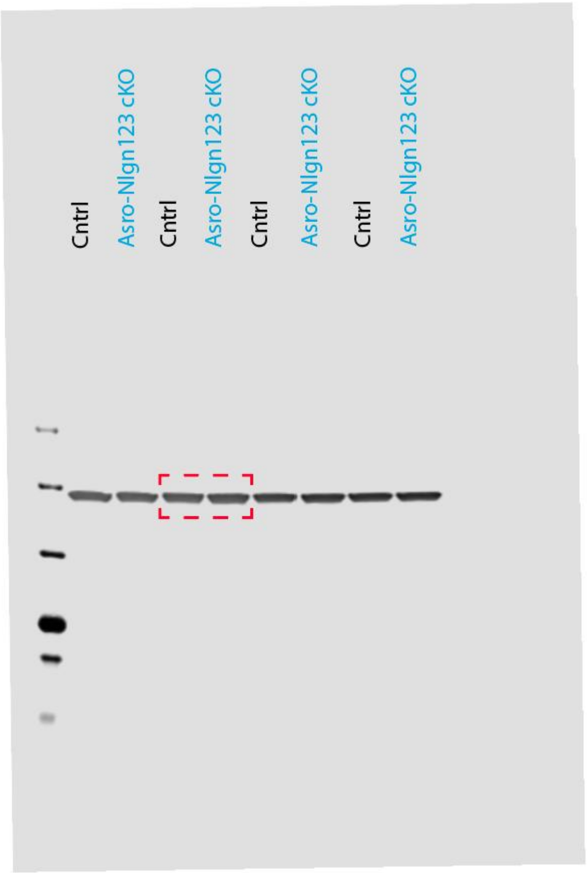

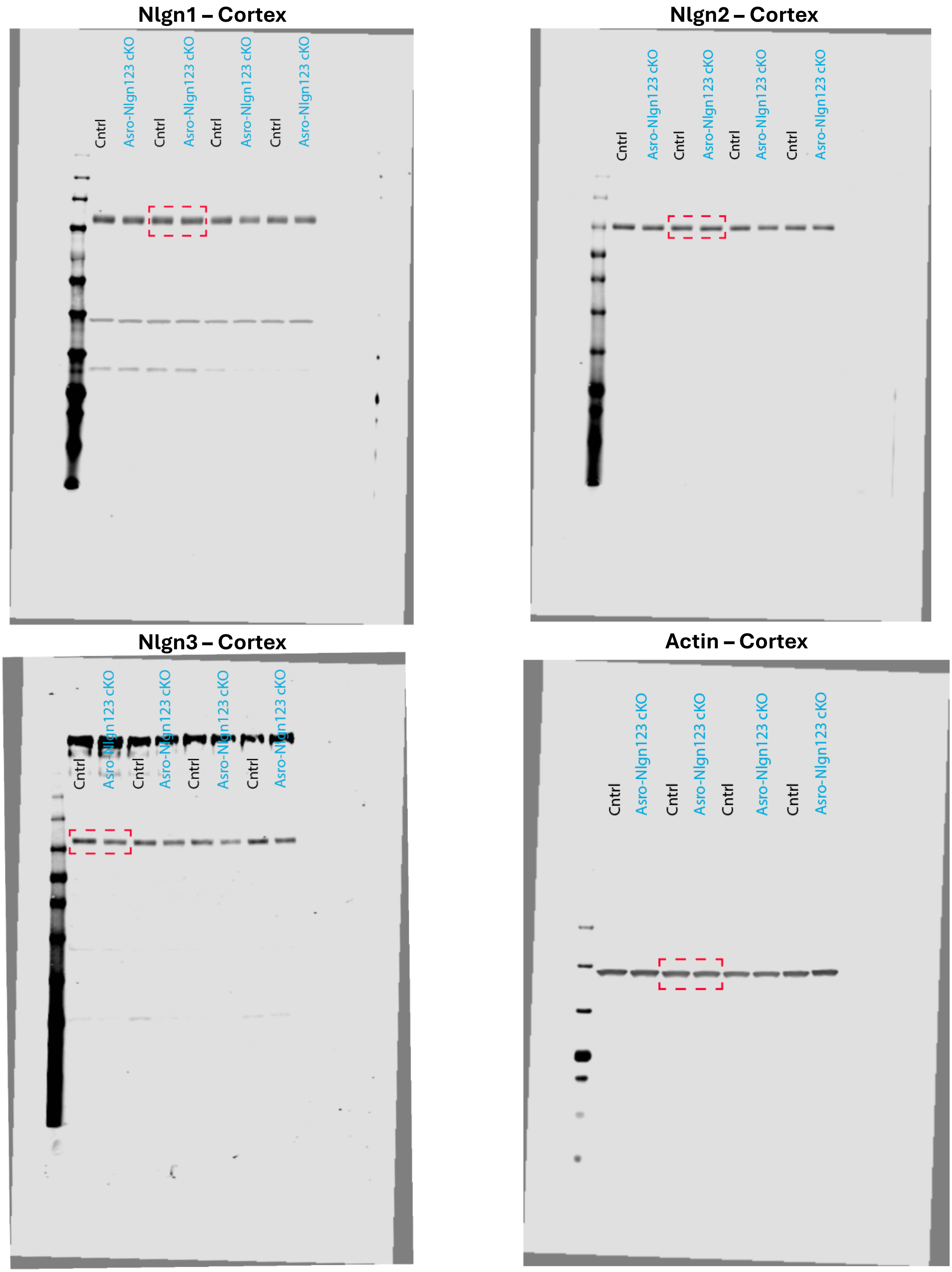

Figure 4, Source Data 5. Original membranes corresponding to Figure 4, panel E. Red dashed boxes indicate the regions cropped for the representative images shown in Figure 4.
