# Supplementary material for: Deletion of neuroligins from astrocytes does not detectably alter synapse numbers or astrocyte cytoarchitecture by maturity
Source: eLife. 2026 Aug 4;12:RP87589. doi: 10.7554/eLife.87589 (PMC13436961; doi:10.7554/eLife.87589)
Supplement: Supplementary file 2 [file elife-87589-fig4-data1.zip › Figure_4_-_source_data_3.pdf]

VGluT2 – Hippocampus

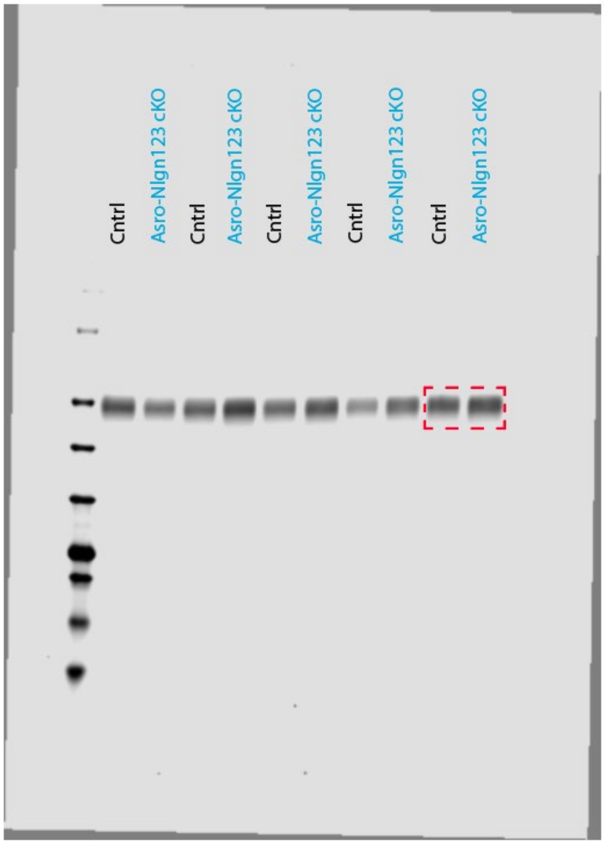

VGluT1 - Hippocampus

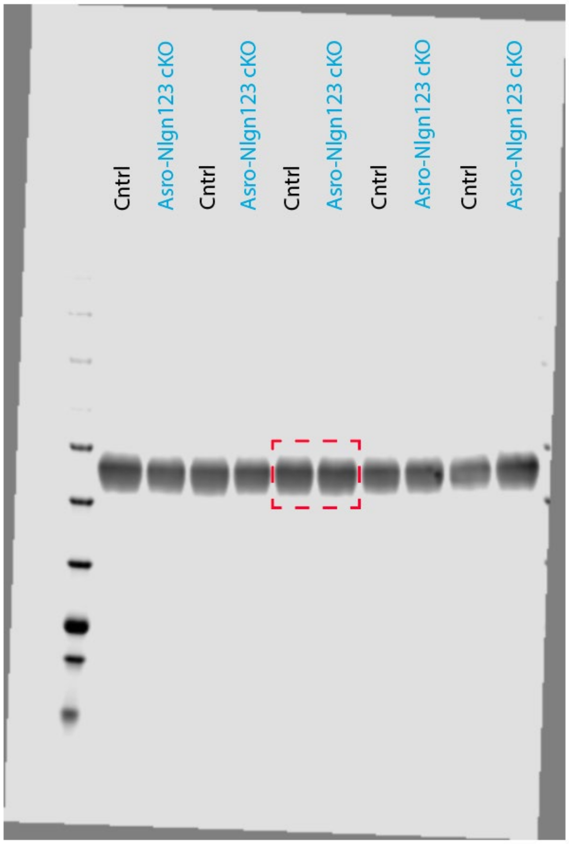

Syt1 - Hippocampus

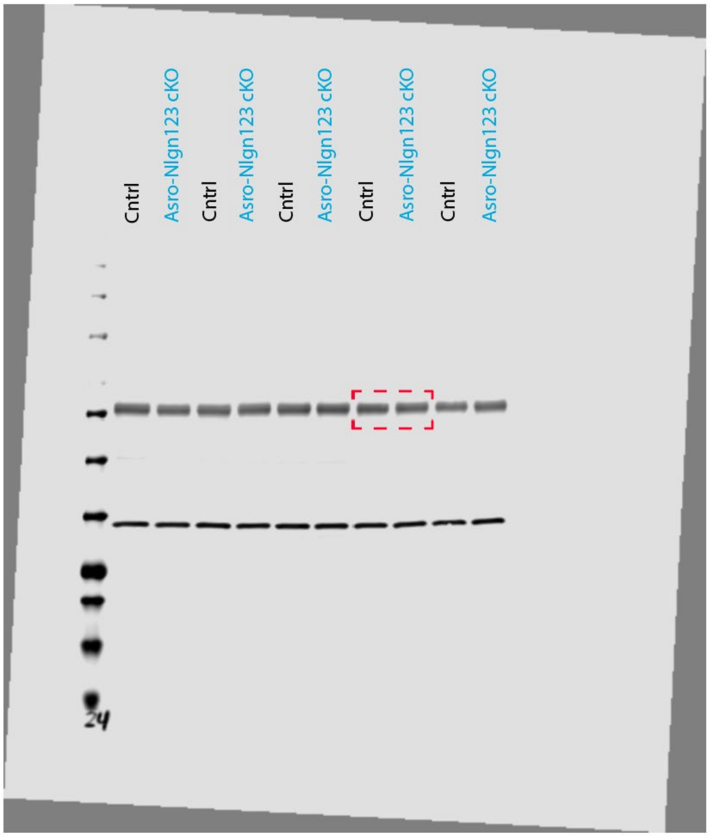

Syb2 - Hippocampus

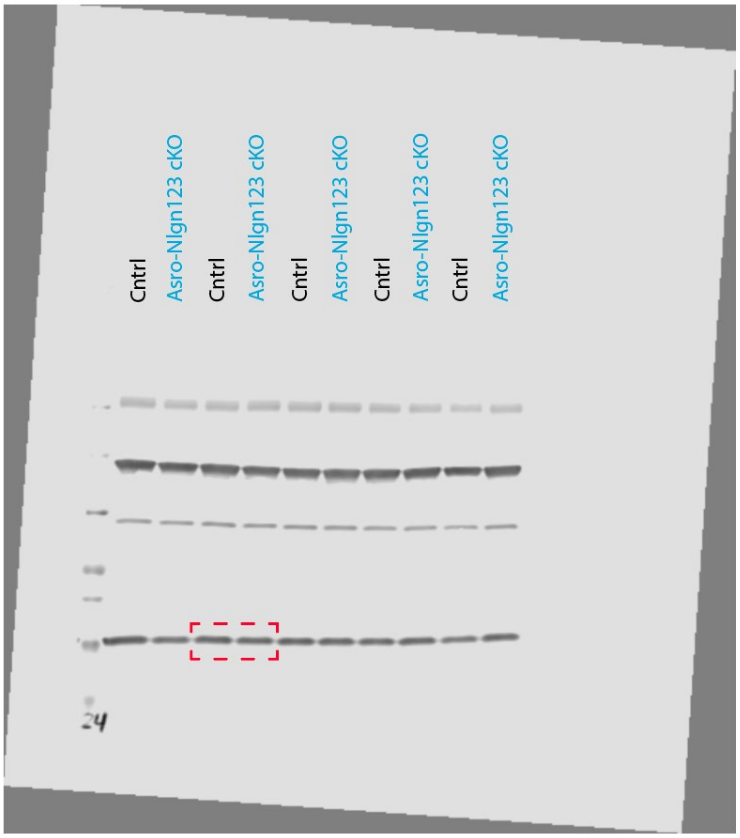

SNAP25 – Hippocampus

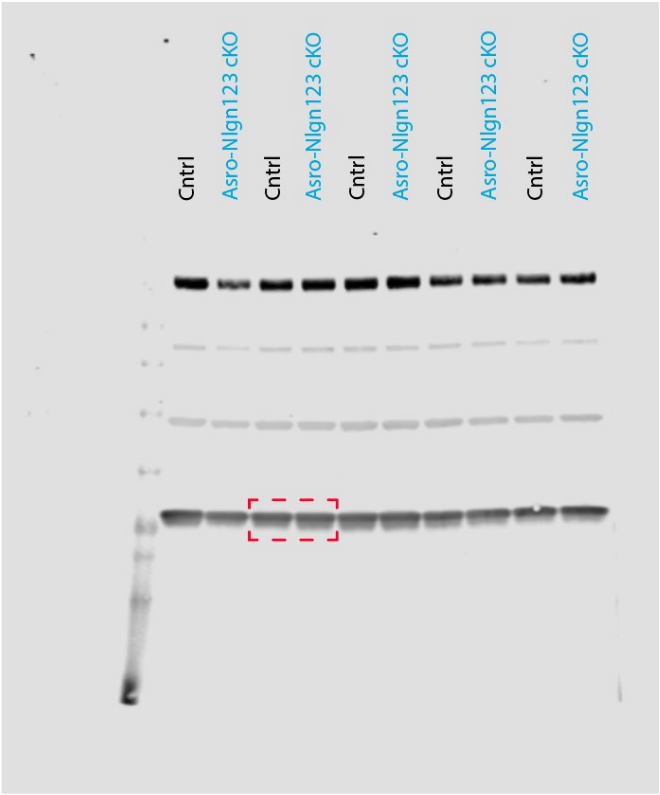

PSD95 – Hippocampus

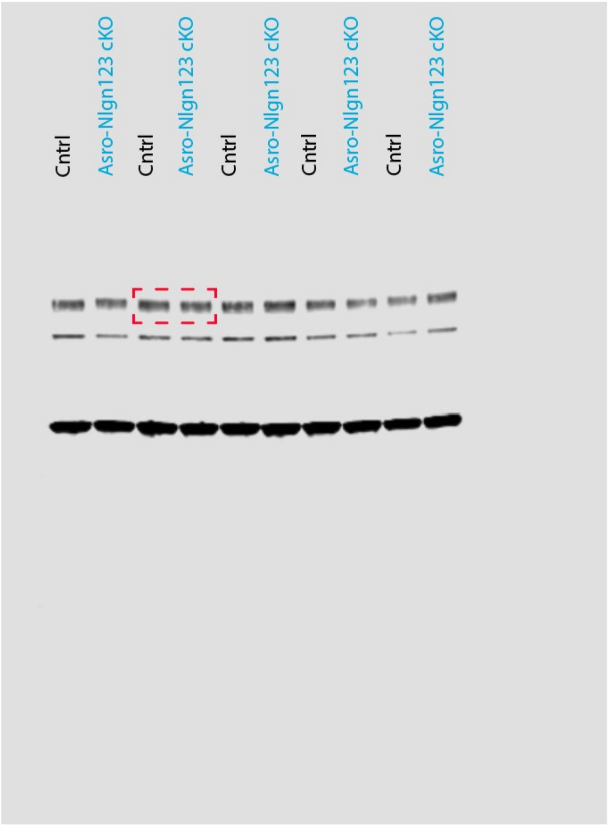

Mint1 – Hippocampus

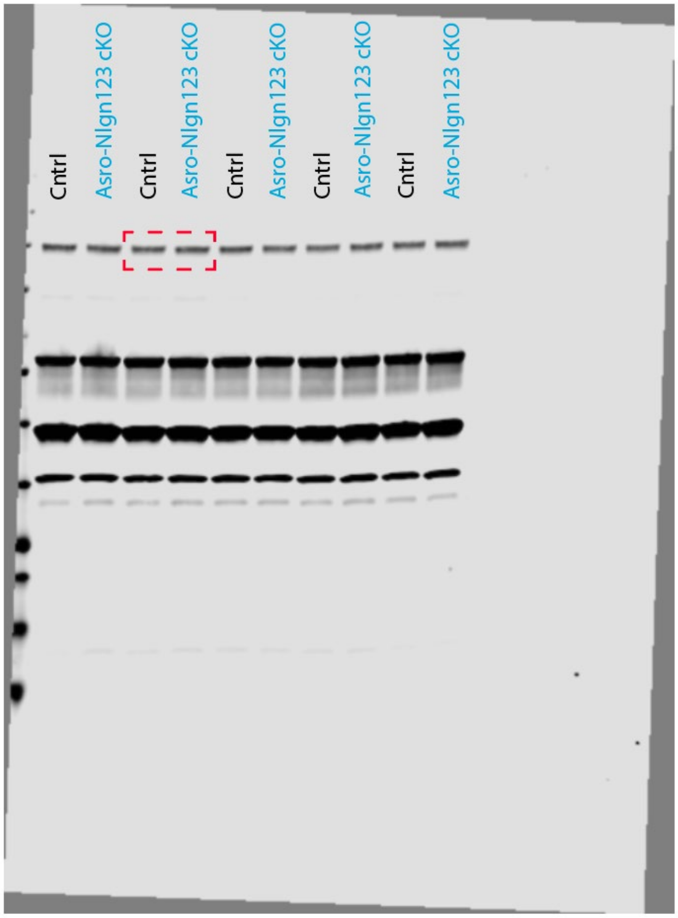

GRIP – Hippocampus

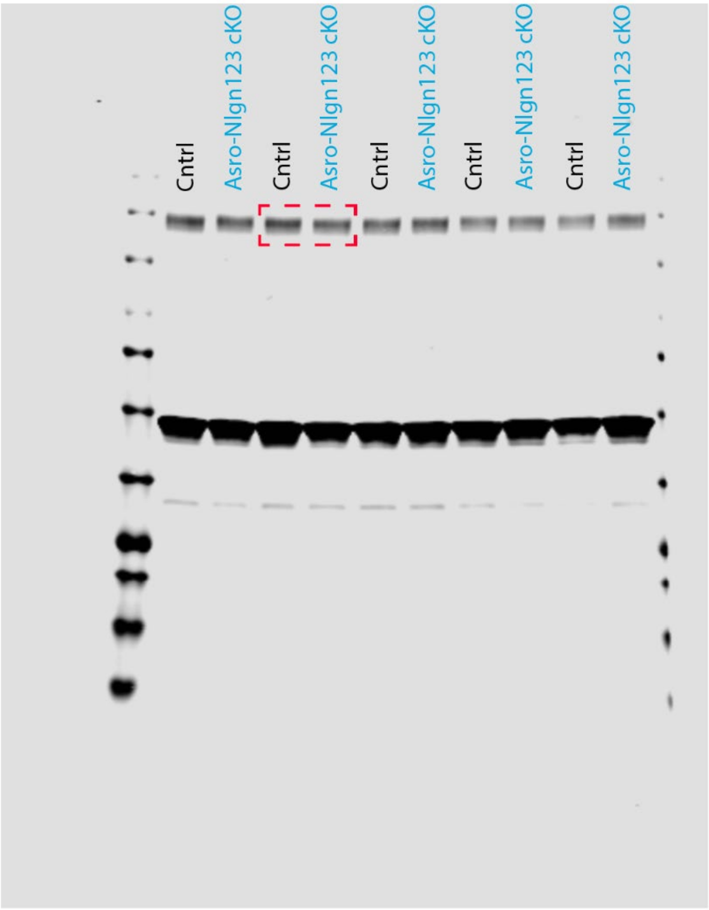

GluR2 – Hippocampus

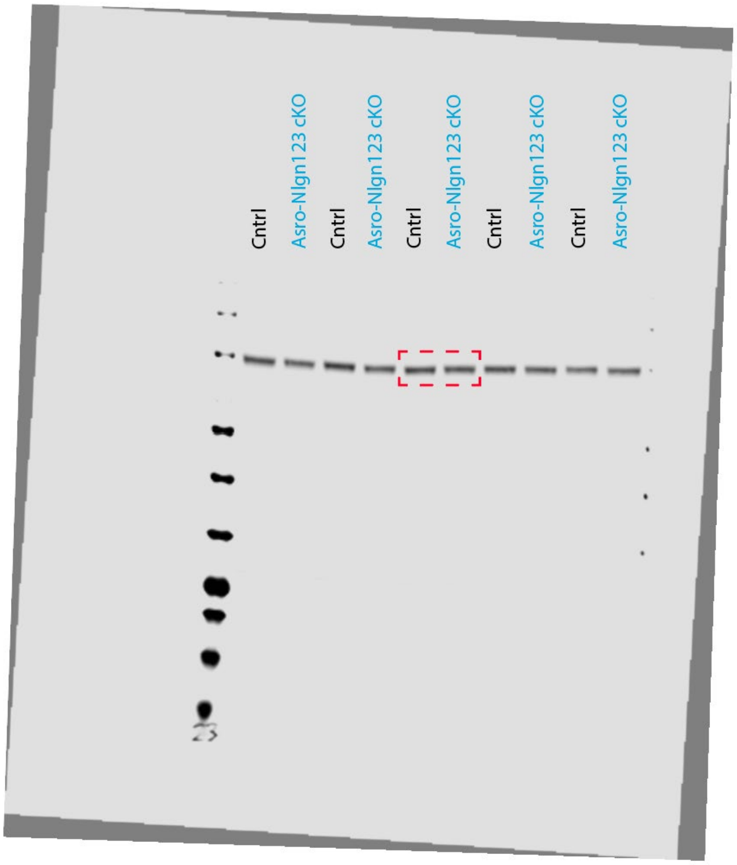

GluR1 – Hippocampus

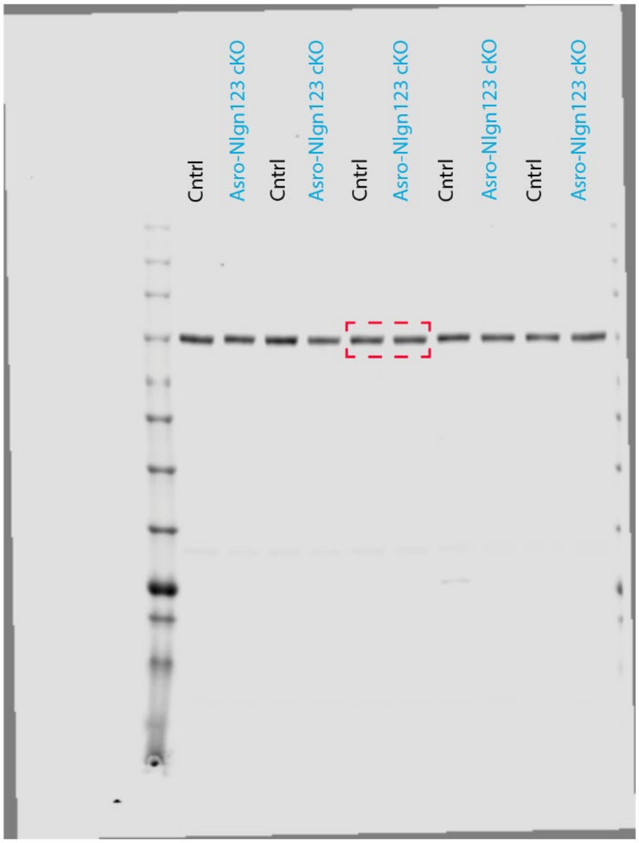

Gephyrin – Hippocampus

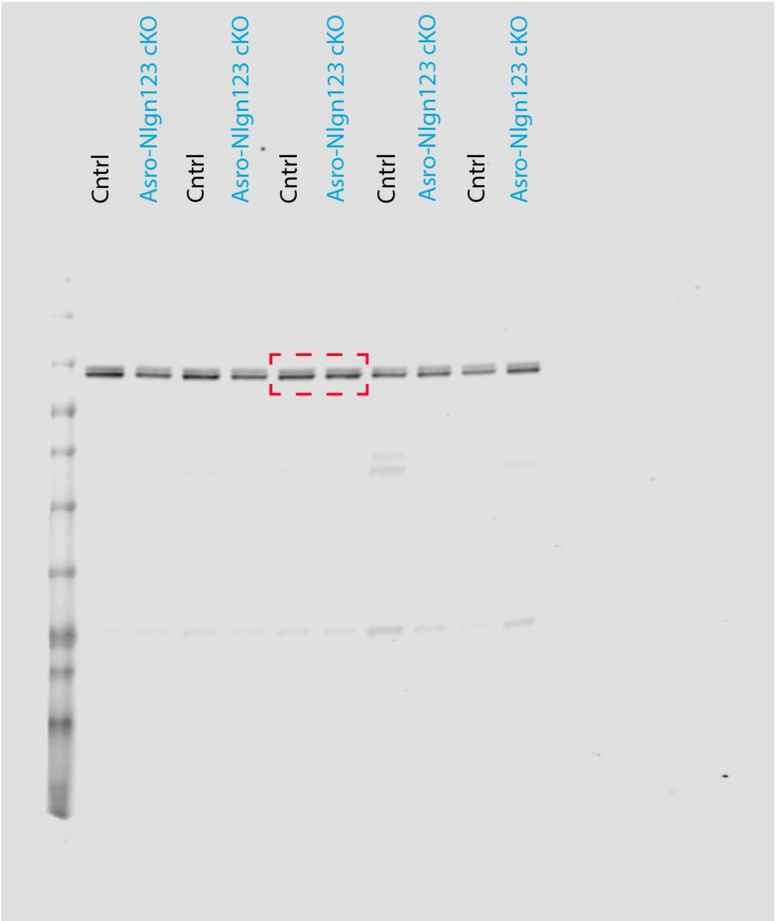

CASK – Hippocampus

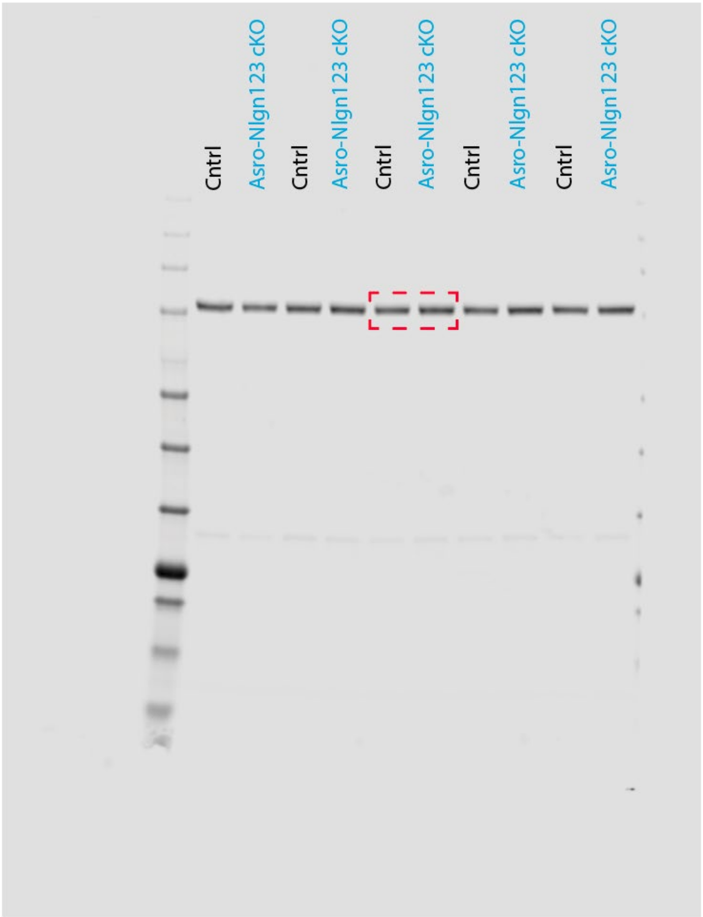

Calbindin – Hippocampus

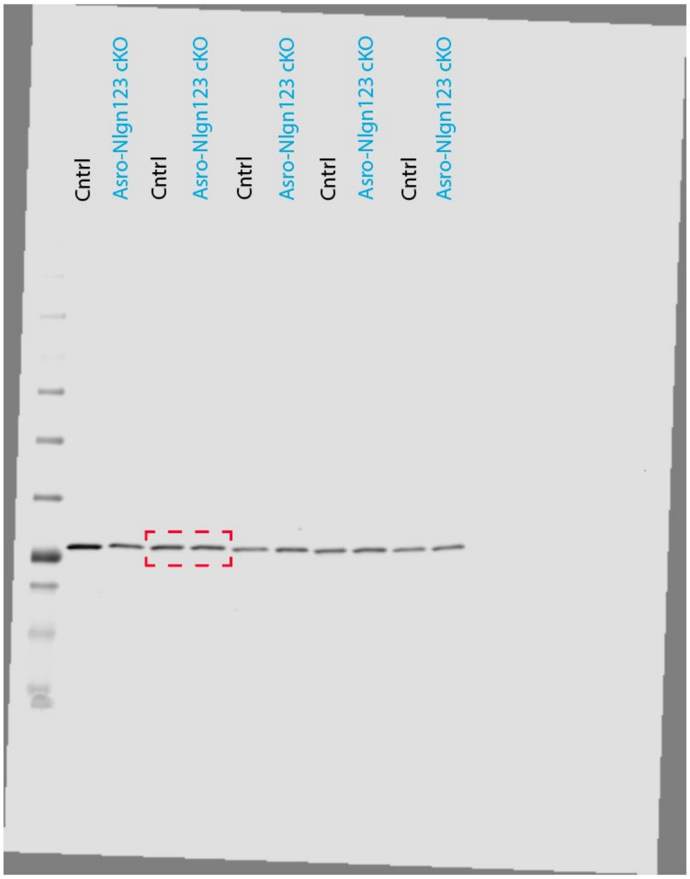

Actin – Hippocampus

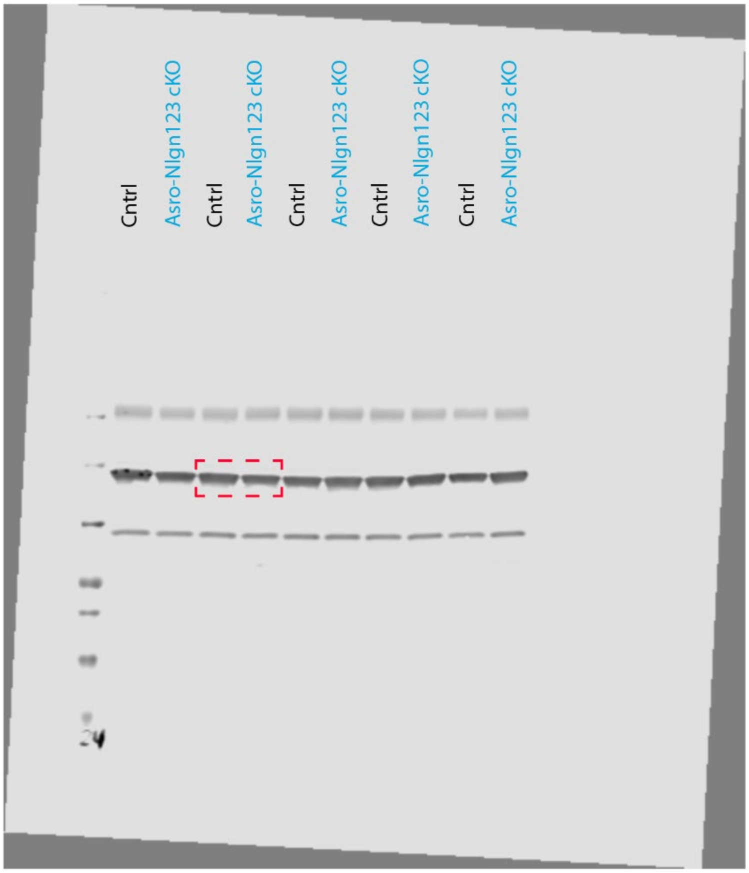

VGluT2 – Cortex

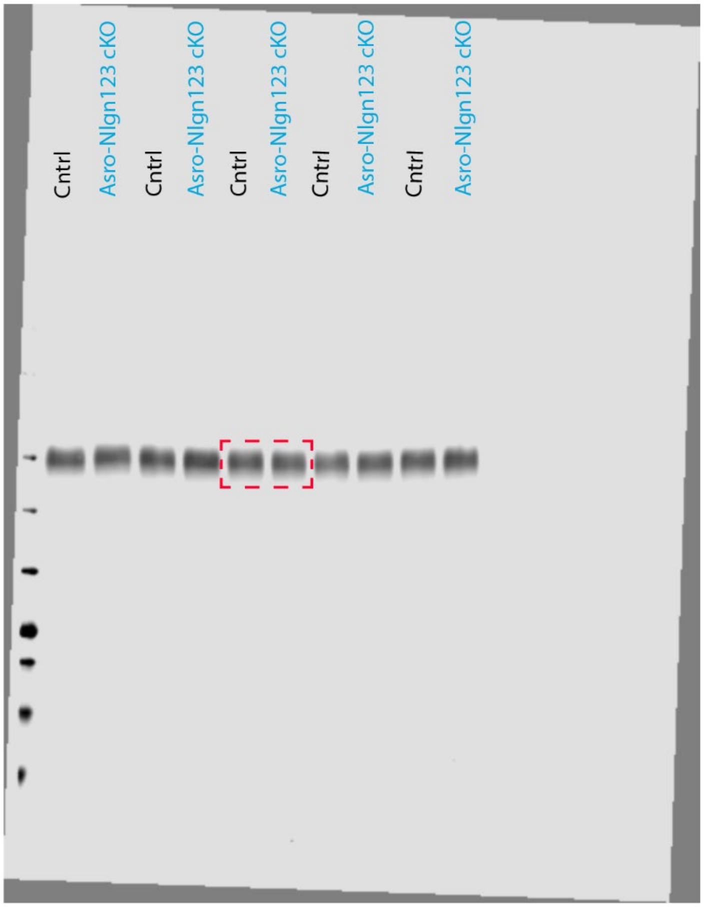

VGluT1 – Cortex

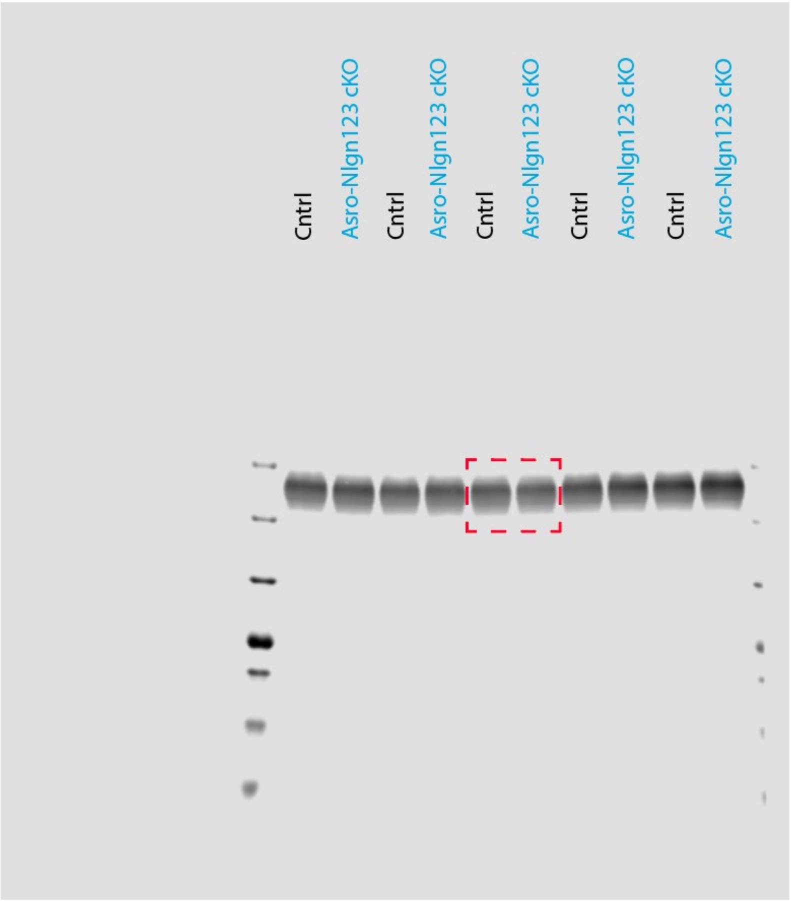

Syt1 - Cortex

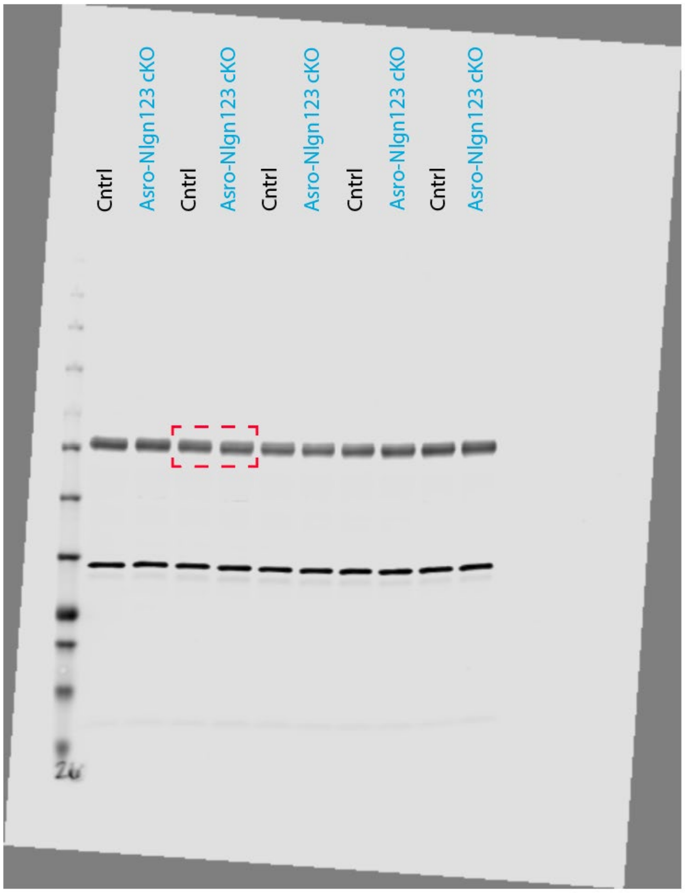

Syb2 - Cortex

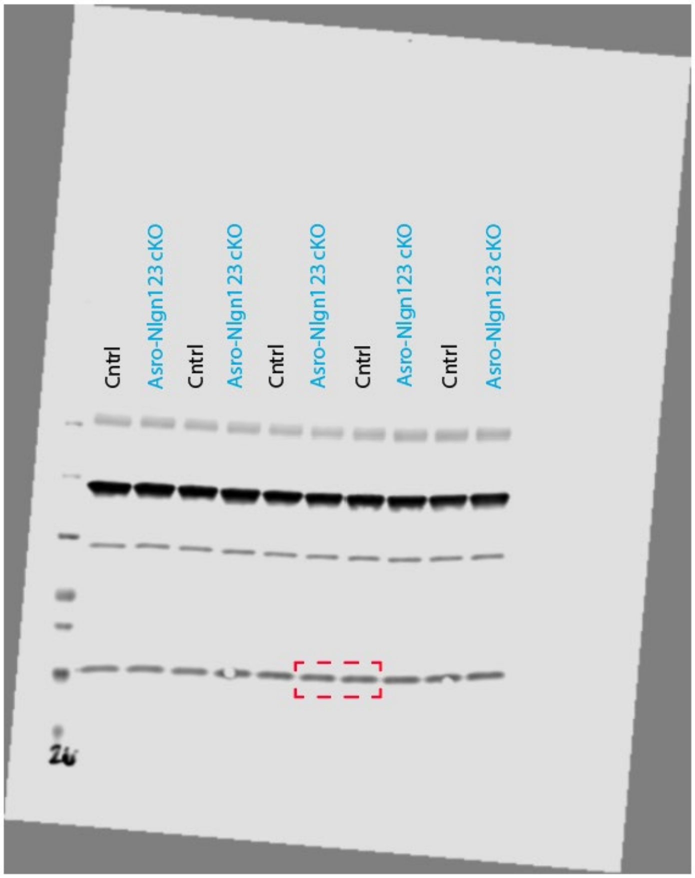

SNAP25 - Cortex

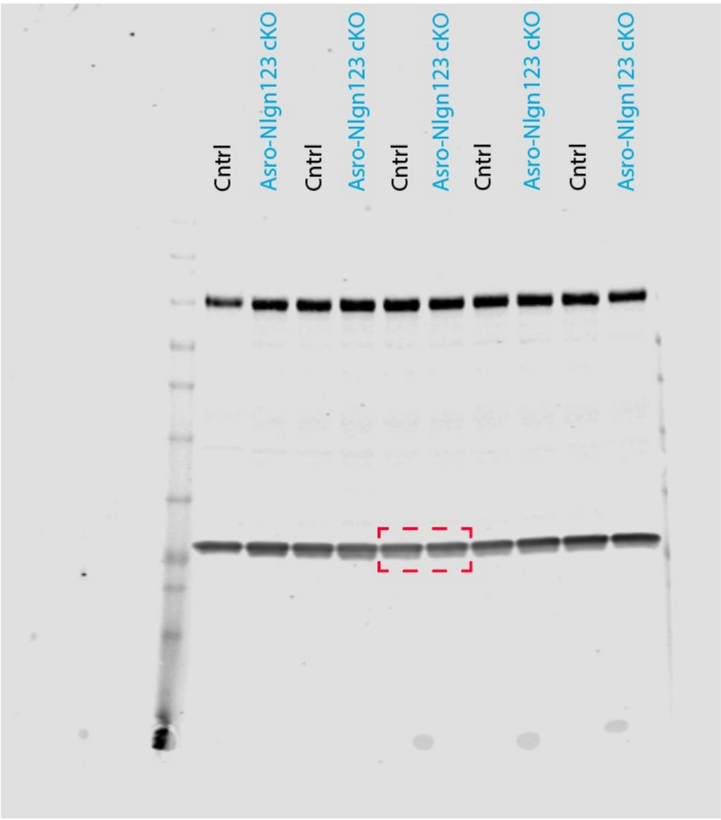

PSD95 - Cortex

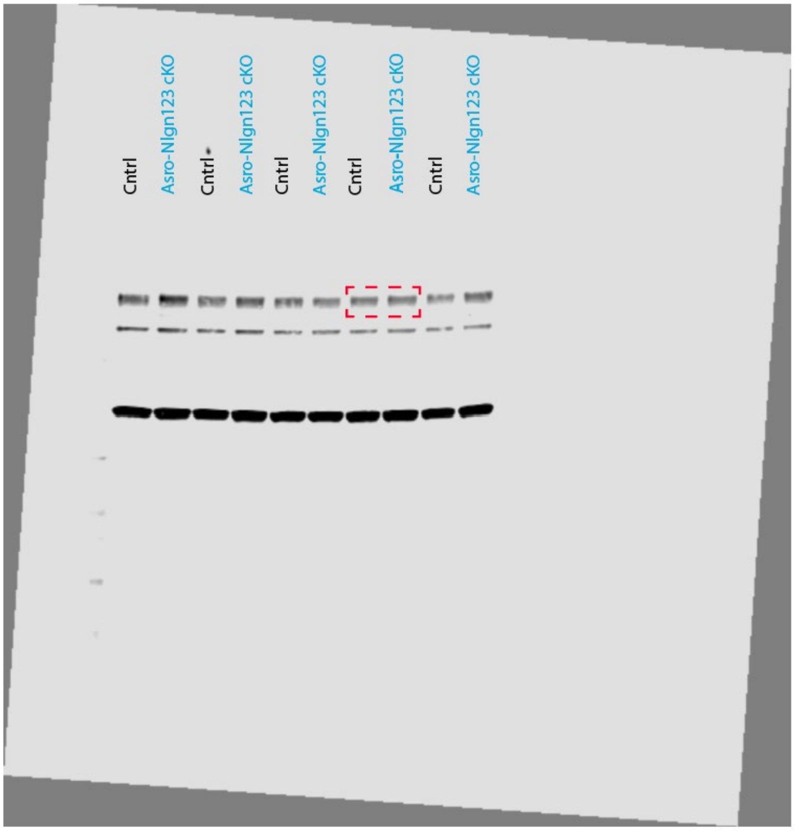

Mint1 – Cortex

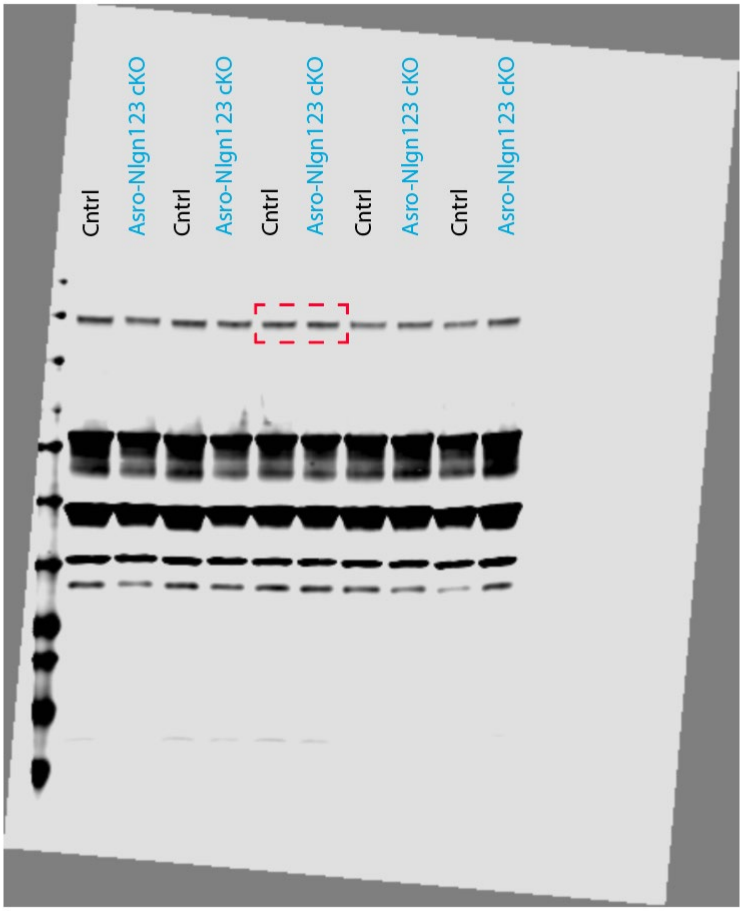

GRIP – Cortex

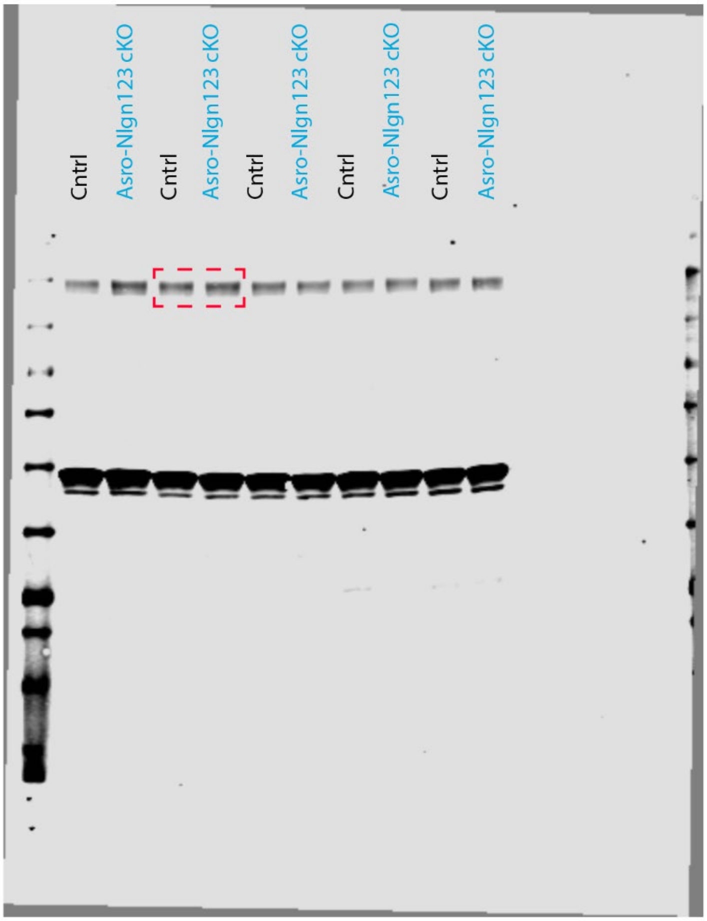

GluR2 – Cortex

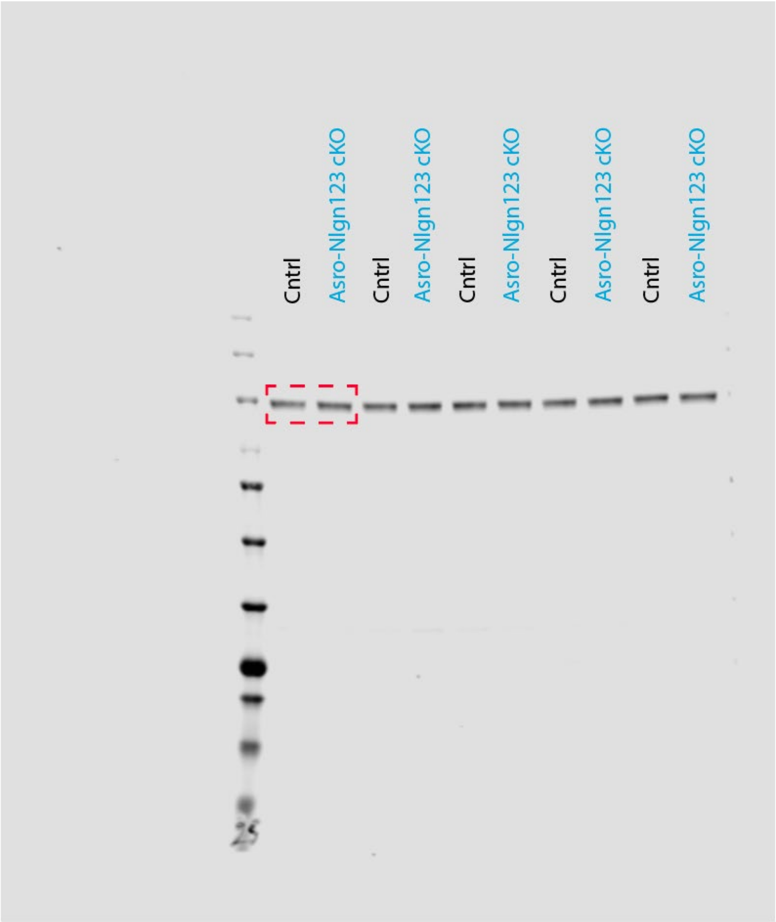

GluR1 – Cortex

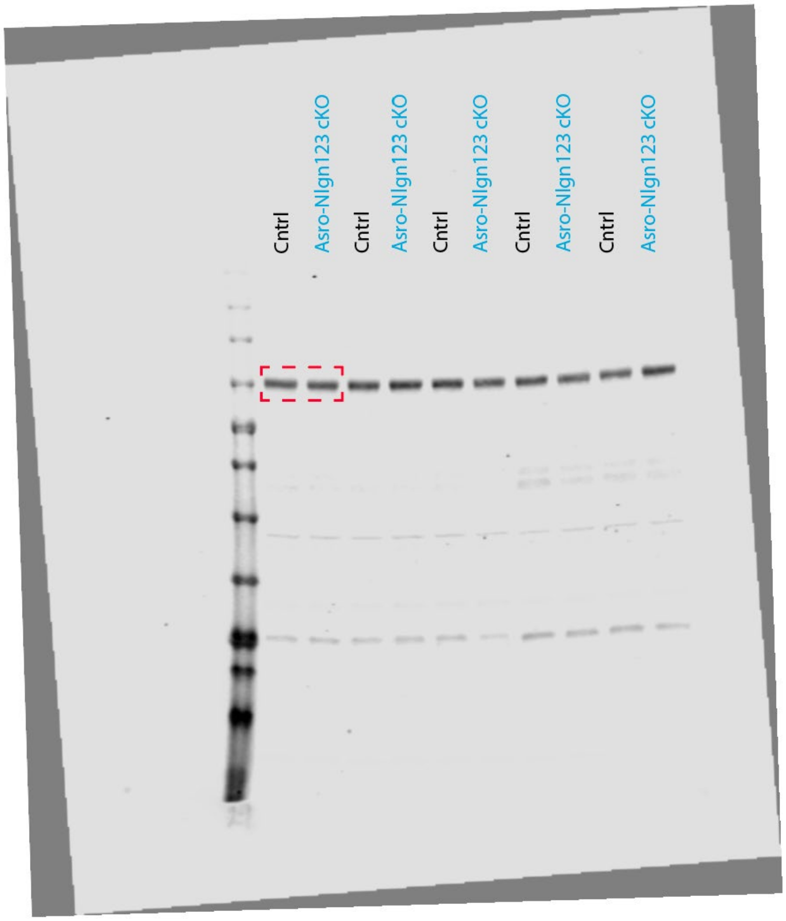

Gephyrin – Cortex

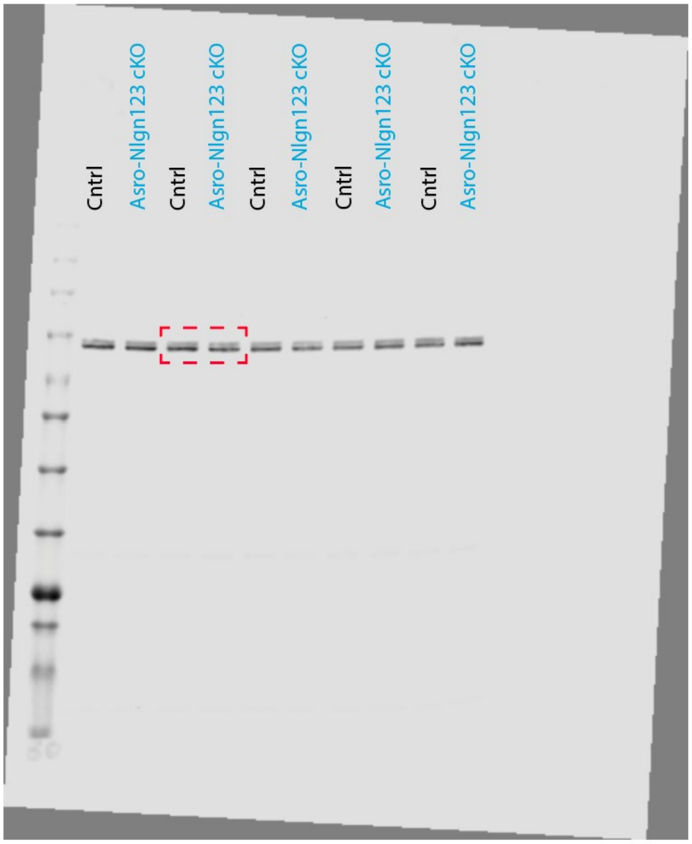

CASK - Cortex

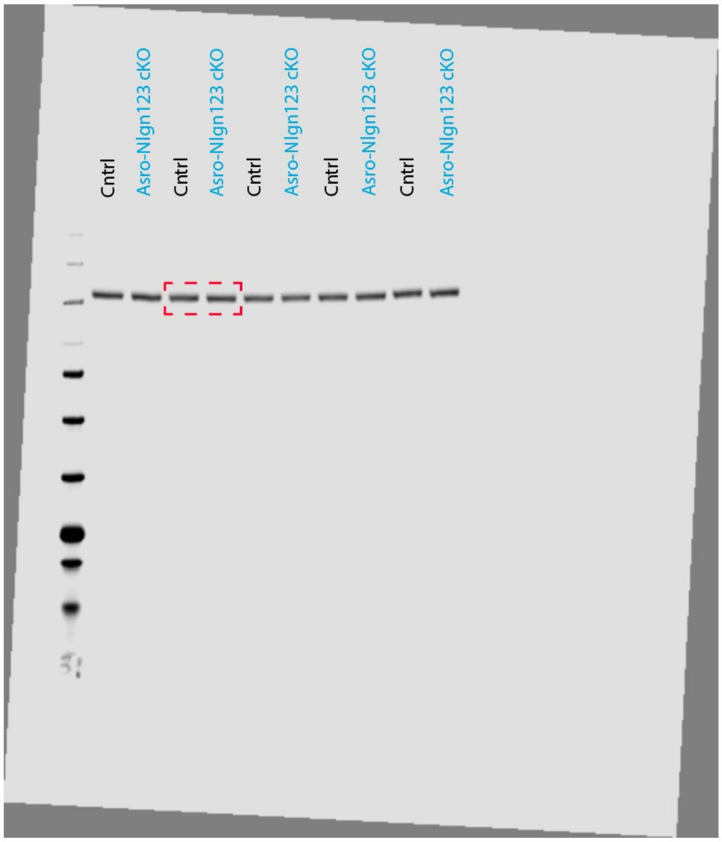

Calbindin – Cortex

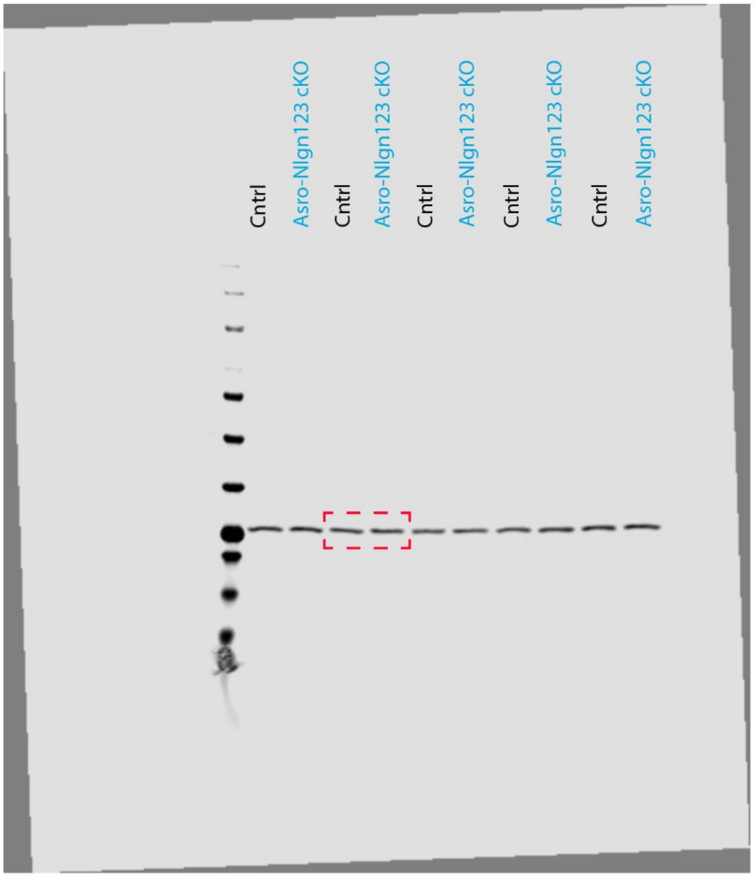

Actin – Cortex

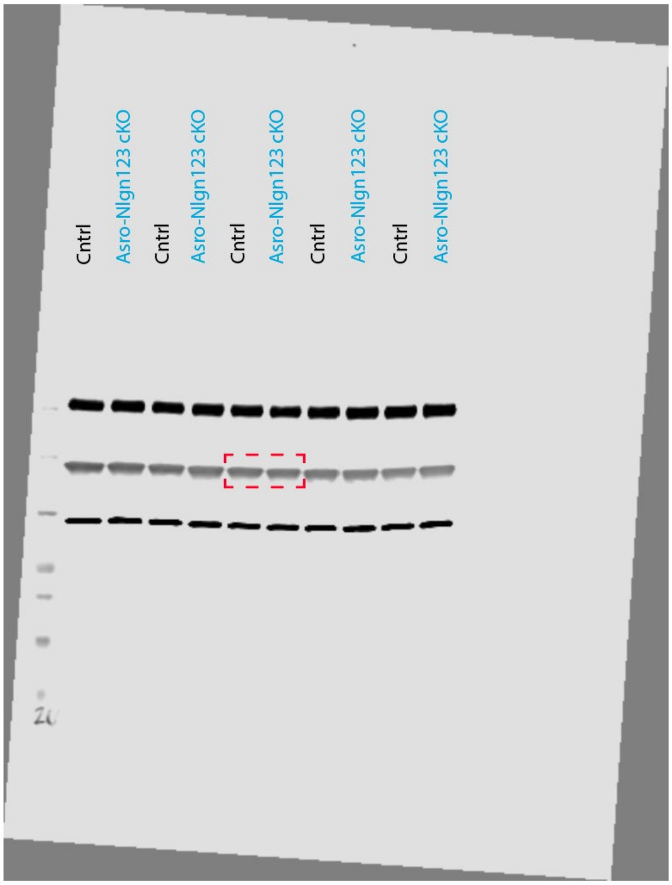

Figure 4, Source Data 3. Original membranes corresponding to Figure 4, panel C. Red dashed boxes indicate the regions cropped for the representative images shown in Figure 4.
