# Supplementary material for: Deletion of neuroligins from astrocytes does not detectably alter synapse numbers or astrocyte cytoarchitecture by maturity
Source: eLife. 2026 Aug 4;12:RP87589. doi: 10.7554/eLife.87589 (PMC13436961; doi:10.7554/eLife.87589)
Supplement: Supplementary file 4 [file elife-87589-fig8-data1.zip › Figure_8_-_source_data_1.pdf]

SPARCL1 – Hippocampus

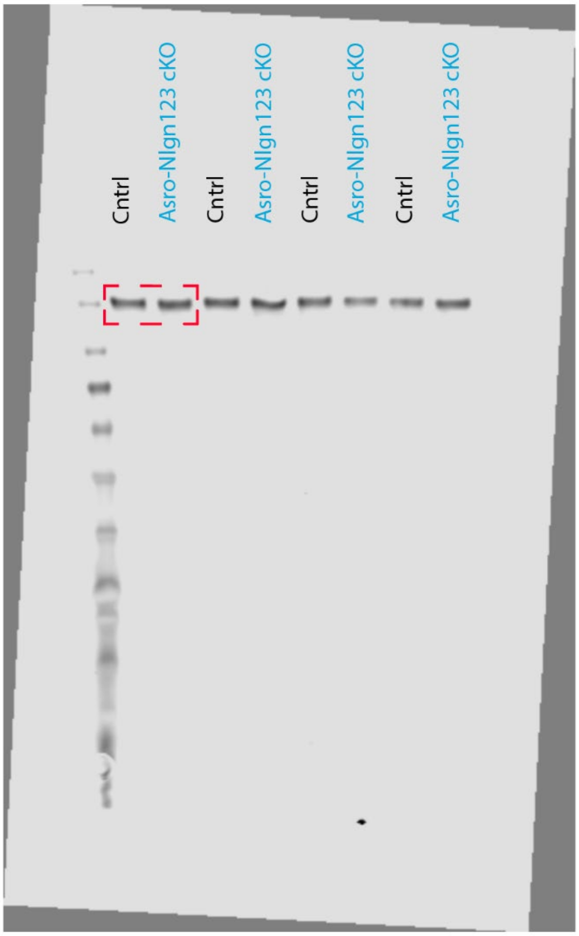

SPARC – Hippocampus

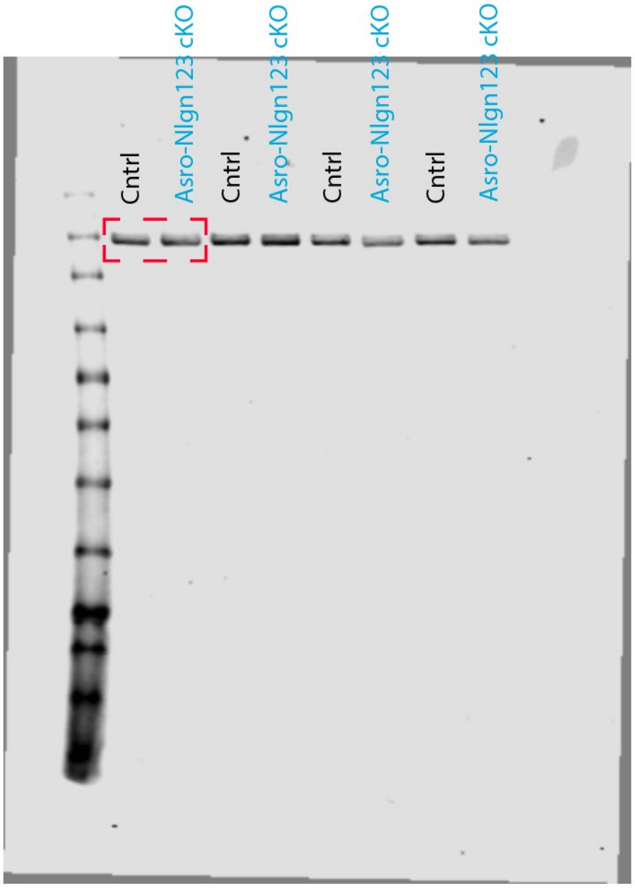

MBP – Hippocampus

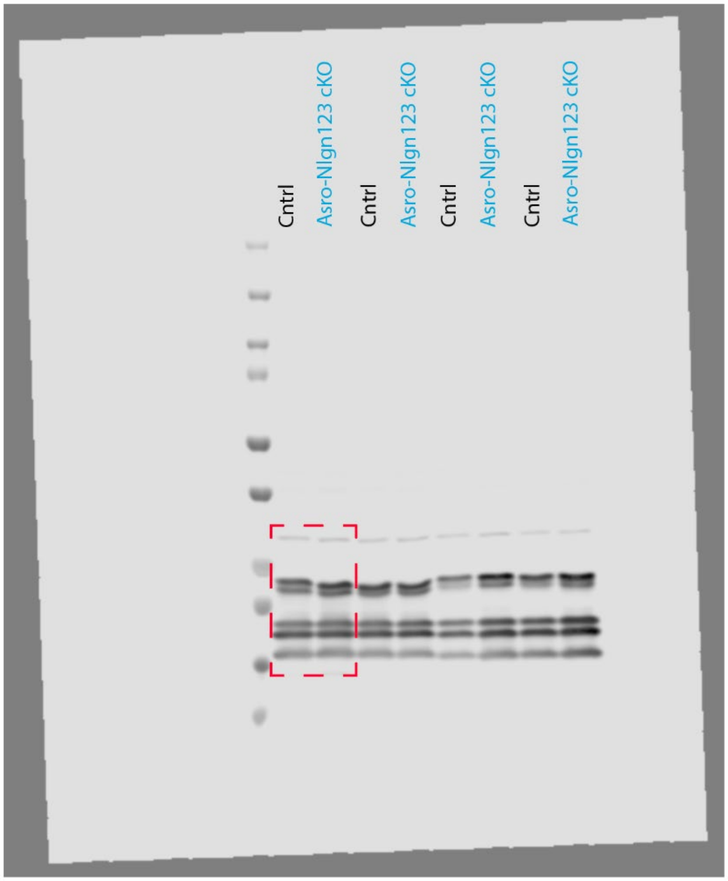

Kir4.1 – Hippocampus

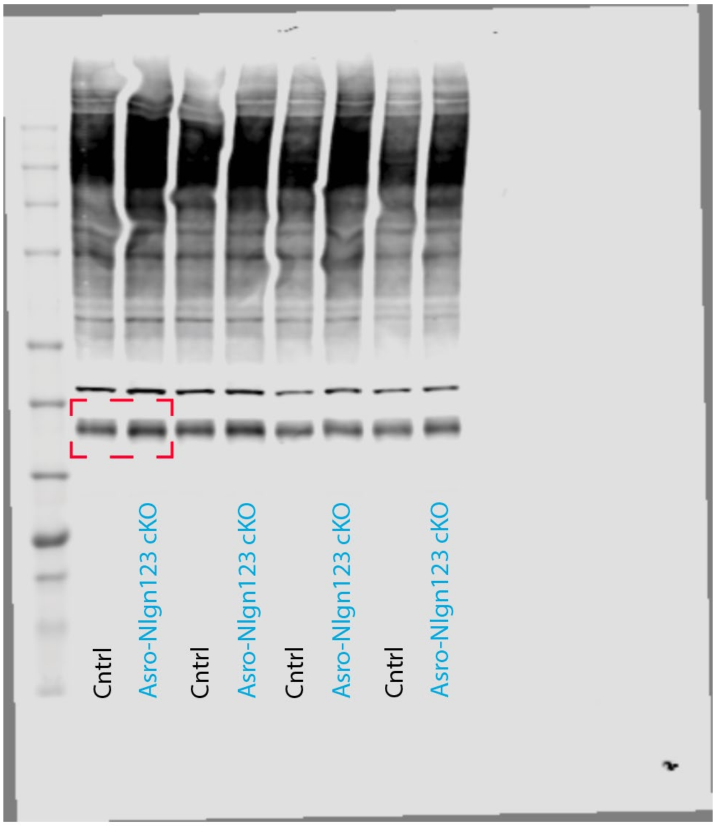

GLAST – Hippocampus

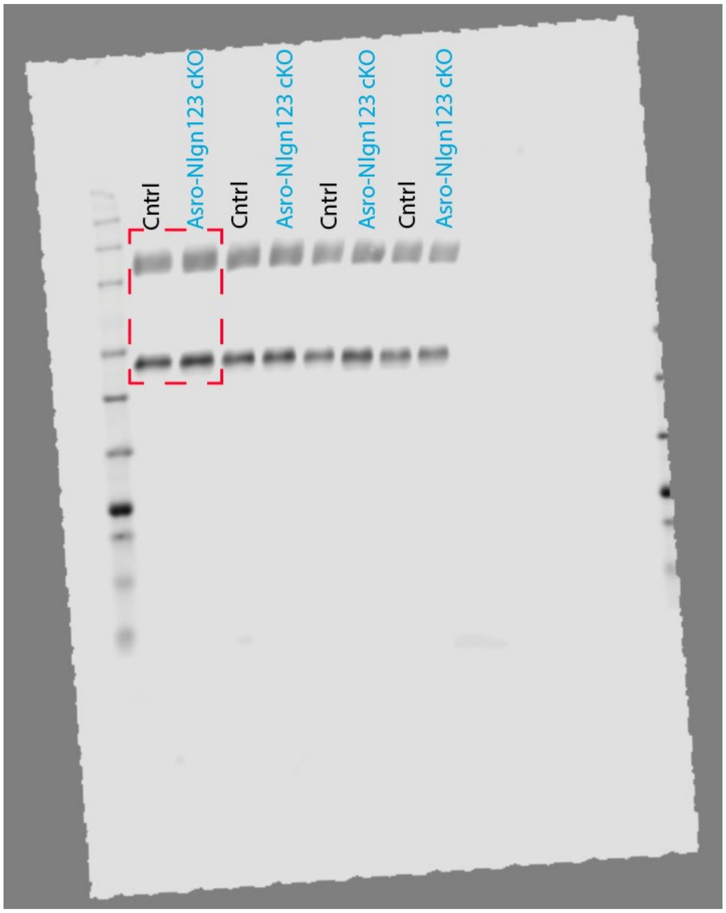

ApoE – Hippocampus

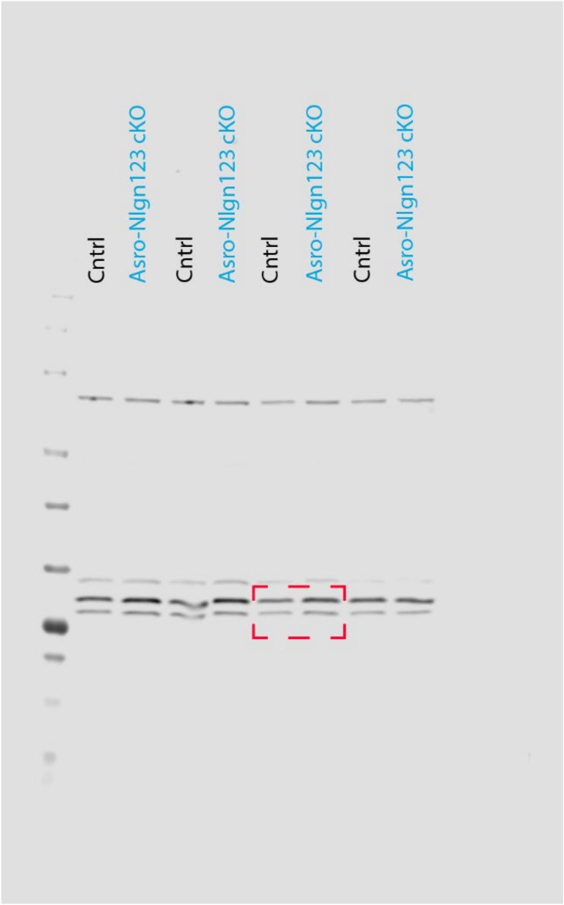

Actin – Hippocampus

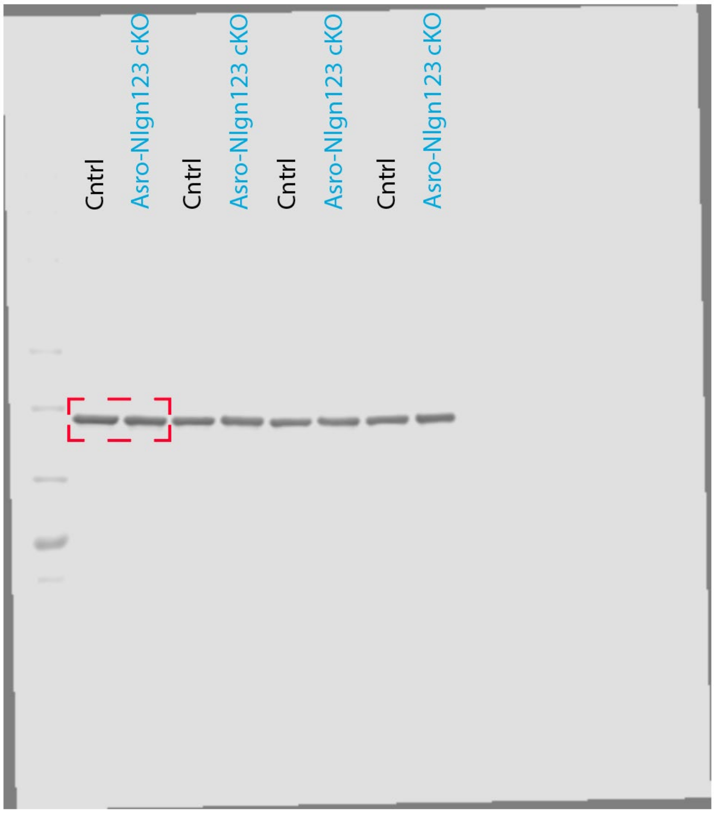

SPARCL1 – Cortex

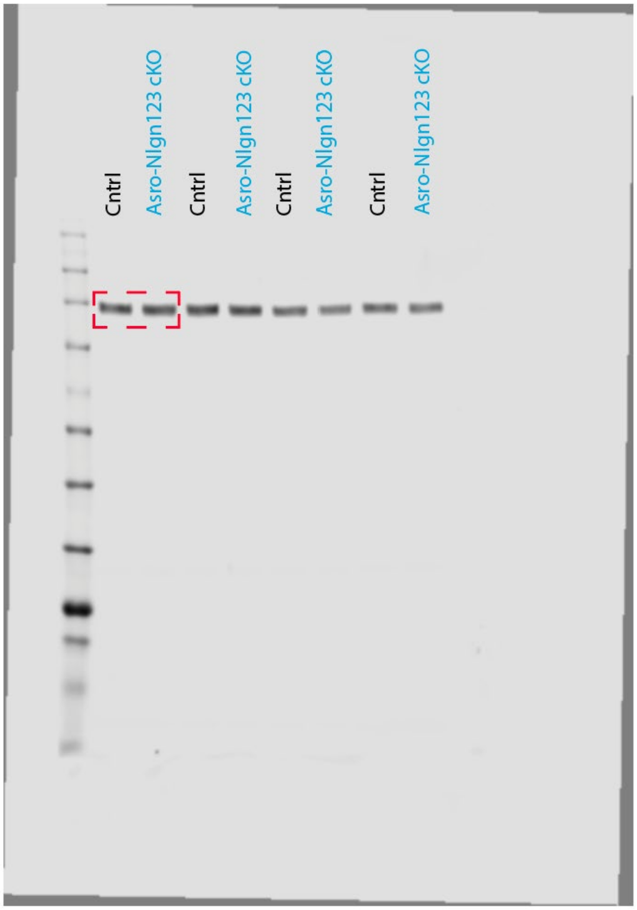

SPARC – Cortex

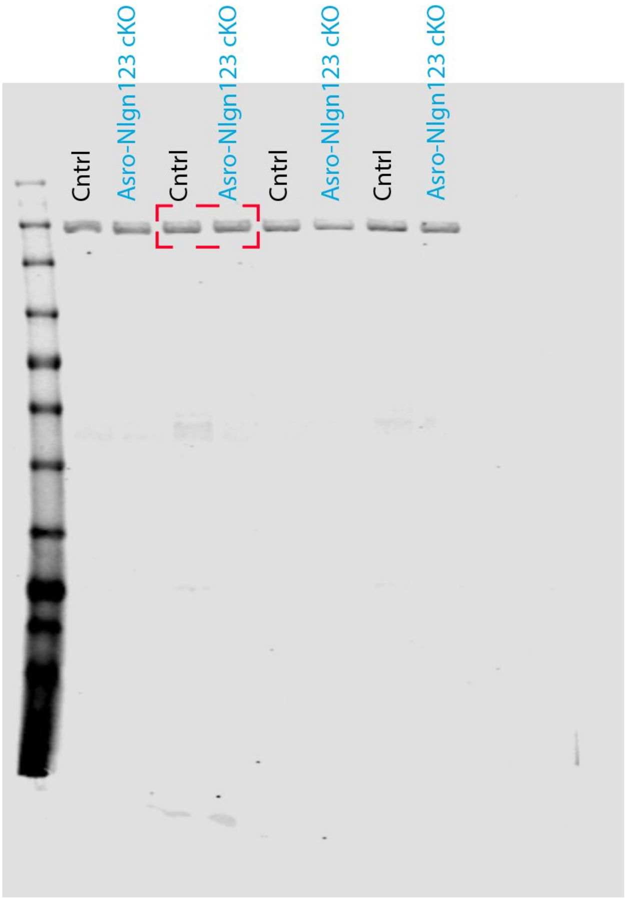

MBP – Cortex

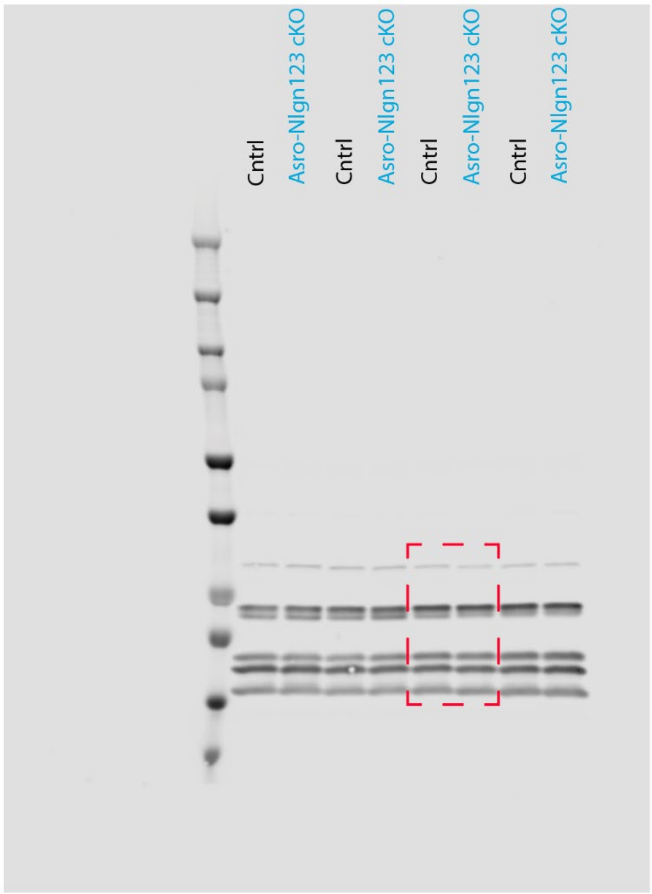

Kir4.1 – Cortex

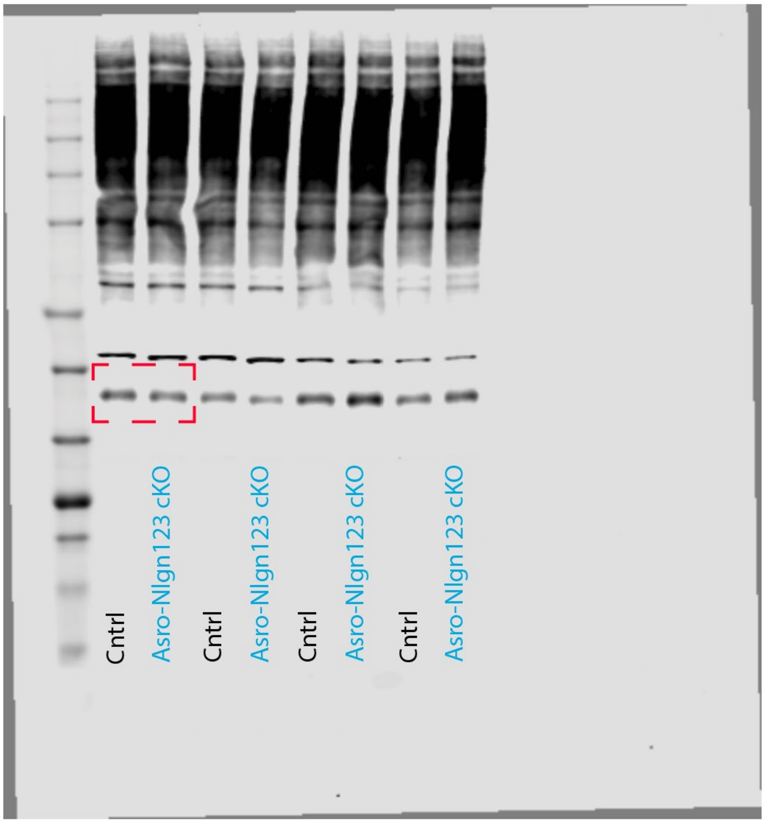

GLAST – Cortex

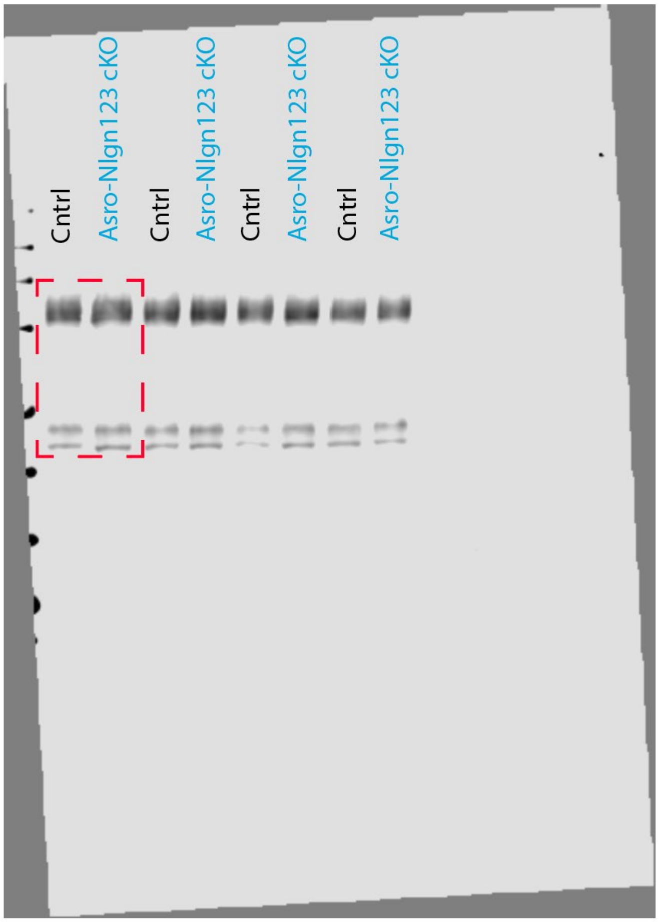

ApoE – Cortex

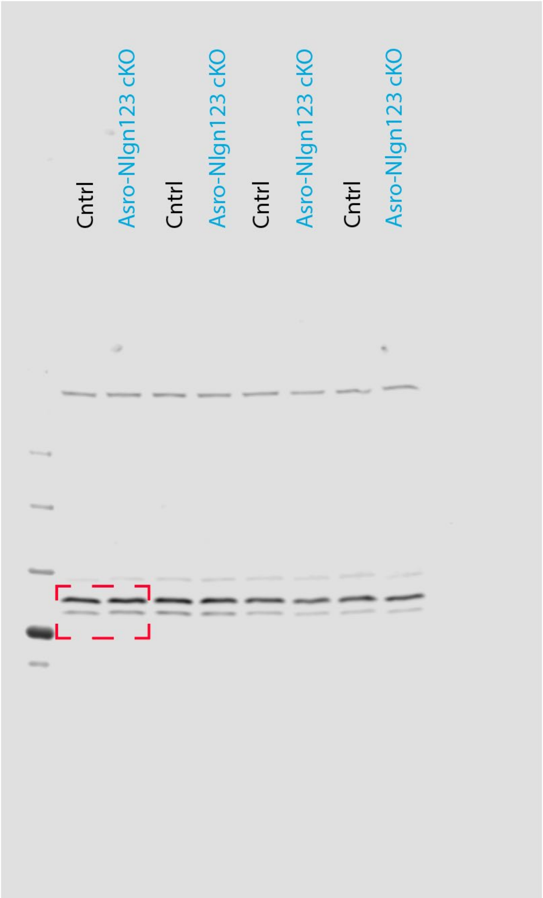

Actin – Cortex

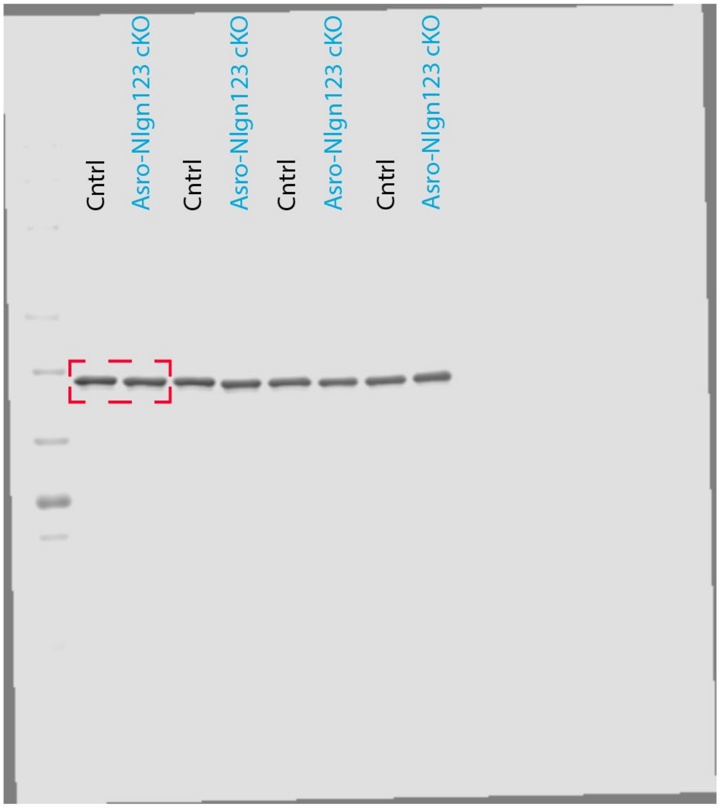

Figure 8, Source Data 1. Original membranes corresponding to Figure 8, panel A. Red dashed boxes indicate the regions cropped for the representative images shown in Figure 8.
