# Supplementary material for: Deletion of neuroligins from astrocytes does not detectably alter synapse numbers or astrocyte cytoarchitecture by maturity
Source: eLife. 2026 Aug 4;12:RP87589. doi: 10.7554/eLife.87589 (PMC13436961; doi:10.7554/eLife.87589)
Supplement: Peer review history

# Editor report: eLife Assessment

**DOI:** 10.7554/eLife.87589.4.sa0

**Authors:** Ma-Li Wong

**Affiliations:** State University of New York Upstate Medical University United States

**Keywords:** Compelling, Fundamental

This **fundamental** study examines whether synaptic cell adhesion molecules neuroligin 1-3 resident on astrocytes, rather than neurons, exert effects on synaptic structure and function. With **compelling** evidence, including rigorous validation of neuroligin deletion efficiency in astrocytes and independent confirmation using human neuron-mouse glia co-cultures, the authors report that deletion of neuroligins 1-3 specifically in astrocytes does not alter synapse formation or astrocyte morphology in the hippocampus or visual cortex. This study provides definitive evidence highlighting the specific role of neuronal neuroligins rather than their astrocytic counterparts in synaptogenesis.

# Referee report: Reviewer #1 (Public review)

**DOI:** 10.7554/eLife.87589.4.sa1

Astrocytes are known to express neuroligins 1-3. Within neurons, these cell adhesion molecules perform important roles in synapse formation and function. Within astrocytes, a significant role for neuroligin 2 in determining excitatory synapse formation and astrocyte morphology was shown in 2017. However, there has been no assessment of what happens to synapses or astrocyte morphology when all three major forms of neuroligins within astrocytes (isoforms 1-3) are deleted using a well characterized, astrocyte specific, and inducible cre line. By using such selective mouse genetic methods, the authors here show that astrocytic neuroligin 1-3 expression in astrocytes is not consequential for synapse function or for astrocyte morphology. They reach these conclusions with careful experiments employing quantitative western blot analyses, imaging and electrophysiology. They also characterize the specificity of the cre line they used. Overall, this is a very clear and strong paper that is supported by rigorous experiments. The discussion considers the findings carefully in relation to past work. This paper is of high importance, because it now raises the fundamental question of exactly what neuroligins 1-3 are actually doing in astrocytes. In addition, it enriches our understanding of the mechanisms by which astrocytes participate in synapse formation and function. The paper is very clear, well written and well illustrated with raw and average data.

Comments on revisions:

My previous comments have been addressed. I have no additional points to make and congratulate the authors.

# Referee report: Reviewer #2 (Public review)

**DOI:** 10.7554/eLife.87589.4.sa2

In the present manuscript, Golf et al. investigate the consequences of astrocyte-specific deletion of Neuroligin (Nlgn) family cell adhesion proteins on synapse structure and function in the brain. Decades of prior research had shown that Neuroligins mediate their effects at synapses through their role in the postsynaptic compartment of neurons and their transsynaptic interaction with presynaptic Neurexins. More recently, it was proposed for the first time that Neuroligins expressed by astrocytes can also bind to presynaptic Neurexins to regulate synaptogenesis (Stogsdill et al. 2017, Nature). However, several aspects of the model proposed by Stogsdill et al. on astrocytic Neuroligin function conflict with prior evidence on the role of Neuroligins at synapse, prompting Golf et al. to further investigate astrocytic Neuroligin function in the current study. Using postnatal conditional deletion of Nlgn1-3 specifically from astrocytes in mice, Golf et al. show that virtually no changes in the expression of synaptic proteins or in the properties of synaptic transmission at either excitatory or inhibitory synapses are observed. Moreover, no alterations in the morphology of astrocytes themselves were found. To further extend this finding, the authors additionally analyzed human neurons co-cultured with mouse glia lacking expression of Nlgn1-4. No difference in excitatory synaptic transmission was observed between neurons cultured in the presence of wildtype vs. Nlgn1-4 conditional knockout glia. The authors conclude that while Neuroligins are indeed expressed in astrocytes and are hence likely to play some role there, this role does not include any direct consequences on synaptic structure and function, in direct contrast to the model proposed by Stogsdill et al.

Overall, this is a strong study that addresses a fundamental and highly relevant question in the field of synaptic neuroscience. Neuroligins are not only key regulators of synaptic function, they have also been linked to numerous psychiatric and neurodevelopmental disorders, highlighting the need to precisely define their mechanisms of action. The authors take a wide range of approaches to convincingly demonstrate that under their experimental conditions, Nlgn1-3 are efficiently deleted from astrocytes in vivo, and that this deletion does not lead to major alterations in the levels of synaptic proteins or in synaptic transmission at excitatory or inhibitory synapses, or in the morphology of astrocytes. The authors have conducted an elegant and compelling analysis demonstrating efficient deletion of astrocytic Nlgn1-3, with deletion rates of 83-96% for Nlgn2 and Nlgn3, and 65-72% for Nlgn1. While the co-culture experiments provide additional support, they are not essential as the in vivo data on astrocytic Nlgn1-3 deletion are compelling on their own. Together, the data from this study provide compelling and important evidence that, whatever the role of astrocytic Neuroligins may be, they do not contribute substantially to synapse formation or function under the conditions investigated.

Comments on revisions:

All of my concerns have been satisfactorily addressed.

The authors have fully addressed my concerns, and have in particular conducted a very elegant and compelling analysis of the degree of deletion of astrocytic Nlgn1-3/4 in their models. This greatly strengthens the main claims of their study and the fundamental nature of their conclusions for the field of synapse biology.

Regarding the co-culture experiments, while I was initially concerned about the lack of controls demonstrating that glia affect synapse formation in human neurons, the authors have appropriately addressed this by clarifying the missing references and explaining that their culture system has been extensively validated in previous studies. Since the data on astrocytic Nlgn1-3 deletion in vivo are compelling on their own, the co-culture experiment provides useful additional support for the main conclusions.

The authors have also added the mouse strain background information to the methods section as requested, which is important for interpreting potential differences with other studies.

# Author comment: Author response

**DOI:** 10.7554/eLife.87589.4.sa3

**Authors:** Samantha Rose Golf, Justin Trotter, Jinzhao Wang, George Nakahara, Xiao Han, Marius Wernig, Thomas Südhof

**Affiliations:** Stanford University Stanford United States; Stanford University Stanford United States; Stanford University Stanford United States; Stanford University Stanford United States; Howard Hughes Medical Institute Palo Alto United States; Stanford University Stanford United States; Stanford University Stanford United States

The following is the authors’ response to the previous reviews

**Public Reviews:**

**Reviewer #1 (Public Review):**

Astrocytes are known to express neuroligins 1-3. Within neurons, these cell adhesion molecules perform important roles in synapse formation and function. Within astrocytes, a significant role for neuroligin 2 in determining excitatory synapse formation and astrocyte morphology was shown in 2017. However, there has been no assessment of what happens to synapses or astrocyte morphology when all three major forms of neuroligins within astrocytes (isoforms 1-3) are deleted using a well characterized, astrocyte specific, and inducible cre line. By using such selective mouse genetic methods, the authors here show that astrocytic neuroligin 1-3 expression in astrocytes is not consequential for synapse function or for astrocyte morphology. They reach these conclusions with careful experiments employing quantitative western blot analyses, imaging and electrophysiology. They also characterize the specificity of the cre line they used. Overall, this is a very clear and strong paper that is supported by rigorous experiments. The discussion considers the findings carefully in relation to past work. This paper is of high importance, because it now raises the fundamental question of exactly what neuroligins 1-3 are actually doing in astrocytes. In addition, it enriches our understanding of the mechanisms by which astrocytes participate in synapse formation and function. The paper is very clear, well written and well illustrated with raw and average data.

Comments on revisions:

My previous comments have been addressed. I have no additional points to make and congratulate the authors.

Thank you for your acceptance.

**Reviewer #2 (Public Review):**

In the present manuscript, Golf et al. investigate the consequences of astrocyte-specific deletion of Neuroligin (Nlgn) family cell adhesion proteins on synapse structure and function in the brain. Decades of prior research had shown that Neuroligins mediate their effects at synapses through their role in the postsynaptic compartment of neurons and their transsynaptic interaction with presynaptic Neurexins. More recently, it was proposed for the first time that Neuroligins expressed by astrocytes can also bind to presynaptic Neurexins to regulate synaptogenesis (Stogsdill et al. 2017, Nature). However, several aspects of the model proposed by Stogsdill et al. on astrocytic Neuroligin function conflict with prior evidence on the role of Neuroligins at synapses, prompting Golf et al. to further investigate astrocytic Neuroligin function in the current study. Using postnatal conditional deletion of Nlgn1-3 specifically from astrocytes in mice, Golf et al. show that virtually no changes in the expression of synaptic proteins or in the properties of synaptic transmission at either excitatory or inhibitory synapses are observed. Moreover, no alterations in the morphology of astrocytes themselves were found. To further extend this finding, the authors additionally analyzed human neurons co-cultured with mouse glia lacking expression of Nlgn1-4. No difference in excitatory synaptic transmission was observed between neurons cultured in the present of wildtype vs. Nlgn1-4 conditional knockout glia. The authors conclude that while Neuroligins are indeed expressed in astrocytes and are hence likely to play some role there, this role does not include any direct consequences on synaptic structure and function, in direct contrast to the model proposed by Stogsdill et al.

Overall, this is a strong study that addresses a fundamental and highly relevant question in the field of synaptic neuroscience. Neuroligins are not only key regulators of synaptic function, they have also been linked to numerous psychiatric and neurodevelopmental disorders, highlighting the need to precisely define their mechanisms of action. The authors take a wide range of approaches to convincingly demonstrate that under their experimental conditions, Nlgn1-3 are efficiently deleted from astrocytes in vivo, and that this deletion does not lead to major alterations in the levels of synaptic proteins or in synaptic transmission at excitatory or inhibitory synapses, or in the morphology of astrocytes. While the co-culture experiments are somewhat more difficult to interpret due to lack of a control for the effect of wildtype mouse astrocytes on human neurons, they are also consistent with the notion that deletion of Nlgn1-4 from astrocytes has no consequences for the function of excitatory synapses. Together, the data from this study provide compelling and important evidence that, whatever the role of astrocytic Neuroligins may be, they do not contribute substantially to synapse formation or function under the conditions investigated.

**Recommendations for the authors:**

**Reviewer #2 (Recommendations for the authors):**

The authors have fully addressed my concerns, and have in particular conducted a very elegant and compelling analysis of the degree of deletion of astrocytic Nlgn1-3/4 in their models. This greatly strengthens the main claims of their study and the fundamental nature of their conclusions for the field of synapse biology.

I am somewhat less convinced by the newly added experiment to investigate deletion of Nlgns1-4 from glia in glia-neuron co-cultures. The authors provide no evidence to show that either WT or cKO glia have any effect on synapse formation or function in human neurons, and therefore, the current lack of a difference could equally result from the fact that both WT and cKO glia were non-functional altogether. The authors cite two studies to state that human neurons do not form synapses in the absence of astrocytes, Zhang et al. 2013 and Huang et al. 2017, but neither seem to be listed in the references (unless Zhang et al. 2014 was meant), making it difficult to assess the relevance of these data. However, since the data on astrocytic Nlgn1-3 deletion in vivo are compelling on their own, I do not see the co-culture experiment as essential for the main conclusions of the study.

Minor comment:

Please add the information on the strain background of the mice to the methods section of the manuscript. Strain background can have a significant impact on many aspects of neuronal function, and this information is therefore essential for the interpretation of potential differences to other studies.

We deeply apologize for forgetting to include the two important references mentioned by the reviewer in the reference list. We understand that the reviewer as a result could not assess the validity of our statement that co-culture of glia is required for efficient synapse formation by human neurons that are induced from ES or iPS cells. Note that this conclusion does not postulate that all synapse formation requires glia, since the cited papers demonstrate that human neurons induced by our protocol still form scarce synapses without glia. This observation has been confirmed in many different experiments that were performed after the data presented in the cited papers. As a result of this extensive prior documentation that human neurons produced by forced expression of Ngn2 require coculture of glia for efficient synapse formation, we do not feel that we need to repeat this basic characterization of our culture system again to validate multiple previous papers and hope the reviewer will concur. We have additionally added the relevant mouse strain information to the methods section.
